# Biotechnological Modulation of Legumes via Fermentation: Impacts on Nutrient Bioaccessibility, Glycemic Index, and Antinutrients—A Scoping Review

**DOI:** 10.3390/foods15142483

**Published:** 2026-07-13

**Authors:** Carolina Noma, Carlos Henrique Pagno, Julio Cesar Colivet Briceno, Priscila Zaczuk Bassinello, Juliana Aparecida Correia Bento

**Affiliations:** 1 Post-Graduate Program in Tropical Agriculture, Federal University of Mato Grosso (UFMT), Avenida Fernando Corrêa Da Costa, 2367, Boa Esperança, Cuiabá 78060-900, MT, Brazil; 2 Food and Nutrition Department, Faculty of Nutrition, Federal University of Mato Grosso (UFMT), Avenida Fernando Corrêa Da Costa, 2367, Boa Esperança, Cuiabá 78060-900, MT, Brazil; carlos.pagno@ufmt.br (C.H.P.); julio.briceno@ufmt.br (J.C.C.B.); 3 EMBRAPA Food and Territories, Rua Cincinato Pinto, Nº 348, Centro, Maceió 57020-050, AL, Brazil; priscila.bassinello@embrapa.br

**Keywords:** solid-state fermentation, lactic acid bacteria, pulses, phytic acid, tannase, predictive glycemic index, in vitro digestion

## Abstract

The global transition toward plant-based diets has driven the inclusion and legumes as primary sources of proteins and micronutrients. However, the raw whole seed hosts complex matrices of antinutritional factors and crystalline starch arrangements that limit proteolytic digestibility, chelate essential minerals, and induce accelerated postprandial glycemic responses. Conventional culinary and thermal treatments applied in isolation are frequently insufficient to disrupt the physicochemical matrix of the seeds, leaving critical gaps regarding how to sustainably optimize mineral bioaccessibility and convert water-soluble starches into stable slowly digestible fractions. This scoping review synthesizes analytical evidence demonstrating that targeted fermentative bioprocessing acts as a microstructural modulator. However, these biochemical outcomes are not unidirectional; the expansion of nutritional value is strictly governed by a complex interplay of substrate properties, process moisture, pH adjustments, and thermal pretreatments in plant defense frameworks and spatially reorganizing starch polymers. Microbial organic acid production and the mechanical penetration of fungal hyphae promote a 90–100% degradation and elimination of phytates and condensed tannins, eliminating non-digestible galacto-oligosaccharides and inactivating trypsin inhibitors. These mechanisms optimize phytate-to-mineral molar ratios, doubling the bioaccessibility of iron, zinc, and calcium in the digestive aqueous phases, while microbial beta-glucosidase expression bioconverts conjugated glycosides into free aglycones with high antioxidant activity. Simultaneously, the induction of molecular retrogradation drives continuous increases in the resistant starch fraction, inducing significant reductions in the hydrolysis index and lowering the predictive glycemic index to low thresholds. These findings consolidate controlled fermentation as a viable biotechnological intervention, providing structural guidelines for the rational design of functional foods, biofortified baked goods, and vegan beverages with high digestive tolerance.

## 1. Introduction

Legumes establish the foundation for sustainable food systems and the development of plant-based diets [1]. Seeds such as chickpea, lentil, pea, fava bean, and cowpea stand out due to their high content of storage proteins, complex carbohydrates, and essential inorganic micronutrients. However, the raw whole seed imposes biological, rheological, and nutritional barriers to human digestion [2]. The cellular architecture of these vegetables hosts a complex network of antinutritional factors, including phytates (phytic acid), condensed and hydrolyzable tannins, saponins, and protease inhibitors. These phytochemical constituents chelate essential divalent and trivalent cations (Fe^3+^, Zn^2+^, Ca^2+^, Mg^2+^), forming insoluble complexes [3]. Consequently, they reversibly or irreversibly inactivate endogenous proteolytic enzymes (pepsin and trypsin), severely depressing the biological value of the diet [4]. Additionally, non-digestible galacto-oligosaccharides from raffinose-family oligosaccharides (RFOs), such as raffinose, stachyose, and verbascose, cause gastrointestinal discomfort, including chronic flatulence and abdominal bloating, due to the natural absence of alpha-galactosidase enzymes in the brush border of the human upper gastrointestinal tract [5].

Beyond these chemical restrictions, starch gelatinization kinetics and crystalline organization dictate accelerated postprandial glycemic responses when legume seeds undergo isolated thermal treatments without prior structural modulation [6]. Thermal processing and baking alter the amorphous fractions of starch, thereby increasing rapidly digestible starch (RDS) and exacerbating blood glucose and insulin spikes in individuals predisposed to cardiometabolic syndromes or type 2 diabetes [7].

In this scenario, fermentation, driven under controlled conditions by lactic acid bacteria (LAB), yeasts, or filamentous fungi, emerges as a non-thermal biotechnological strategy to redesign legume microstructure. Fermentation triggers microbial enzymatic cascades and activates endogenous seed hydrolases via medium acidification [1,8]. This bioprocess reduces antinutritional factors, releases antioxidant phenolic fractions, promotes the proteolysis of storage proteins, and converts starch into refractory slow-digestible starch (SDS) and resistant starch (RS) fractions [6].

Beyond its profound impact on the nutritional and structural architecture of plant-based matrices, the adoption of fermentative bioprocessing offers a compelling techno-economic advantage for the food industry. While conventional physical and chemical treatments often entail high operational costs and energy demands, both solid-state (SSF) and submerged fermentation (SmF) present scalable pathways toward cost-effective production [9]. SSF is characterized by its low capital expenditure (CAPEX), as it utilizes agro-industrial by-products as substrates, effectively reducing raw material costs while requiring less water and energy for downstream processing. From a circular economy perspective, the ability of lactic acid bacteria and filamentous fungi to upcycle low-value legumes into high-value functional ingredients, by eliminating the need for expensive synthetic fortification and reducing energy-intensive thermal stages, positions fermentation as a superior biotechnological intervention. Consequently, the integration of these processes ensures not only the delivery of bioavailable nutrients and low-glycemic responses but also enhances market competitiveness through streamlined resource efficiency and sustainable industrial scalability [10].

Although the current scientific literature acknowledges the benefits of bioprocessing, existing reviews on the fermentation of grains and legumes address the reduction in antinutritional factors, bioaccessibility, and the glycemic index in a fragmented manner. Recent systematic studies have exhaustively mapped the dynamics of antinutrient reduction and the enhancement of nutritional bioavailability driven by fermentation in these matrices [11,12,13]. In contrast, research focused on the glycemic aspect is limited to evaluating glycemic control resulting from habitual legume consumption, without considering the specific physical and biochemical modulations induced by the fermentation process [14,15]. This gap in the literature highlights that, while the enzymatic degradation of antinutritional complexes is well documented, the simultaneous impact of these structural changes on starch digestibility and postprandial glycemic response still lacks a structured synthesis. Furthermore, recent analyses of global fermented foods report limitations in describing the mechanisms and fermentation variables of legumes regarding glucose regulation [16]. Therefore, the novel contribution of this scoping review lies in filling this fundamental gap, positioning itself as the first study to simultaneously integrate data on glycemic index modulation, the mitigation of antinutritional barriers, and nutrient bioaccessibility, aiming to address the emerging need for the techno-functional optimization of these plant-based matrices. To address this gap and define the state of the art based on the PCC (Population, Concept, Context) mnemonic, this scoping review aims to answer the following research question: ‘How does legume fermentation modulate the glycemic index, reduce antinutritional factors, and influence nutrient bioaccessibility?’

## 2. Methodology

This scoping review was conducted in accordance with the Joanna Briggs Institute (JBI) methodology for scoping reviews [17] and followed the Preferred Reporting Items for Systematic Reviews and Meta-Analyses extension for Scoping Reviews (PRISMA-ScR) checklist guidelines [18,19].

### 2.1. Review Question

The research question was formulated using the PCC mnemonic (Population, Concept, and Context). Grains (legumes and pulse) were established as the study Population (P); Fermentation (bioprocessing by lactic acid bacteria, yeasts, or fungi) served as the Concept (C); and glycemic index modulation (glycemic response and starch digestibility), antinutrient reduction, and bioaccessibility of nutrients/bioactive compounds constituted the Context (C). Accordingly, the final research question was: ‘How does the fermentation of grains (legumes and pulse) influence the modulation of the glycemic index, the reduction in antinutritional factors, and the bioaccessibility of nutrients?’

### 2.2. Search Strategy

The search strategy followed the three-step process recommended by the Joanna Briggs Institute (JBI): Step 1—An initial preliminary search was conducted in PubMed and Scopus to identify keywords and index terms (MeSH/Emtree) located in the titles and abstracts of relevant articles. Step 2—The identified keywords and index terms were combined into comprehensive search strings using Boolean operators (AND/OR) and adapted for each selected database: Web of Science (abstract search), LILACS/VHL (title, abstract, and keyword search), PubMed (title/abstract search), and Scopus (title, abstract, and keyword search). The search was limited to publications from the last 10 years, with no language restrictions applied. Step 3—The reference lists of all included studies were hand-searched to identify additional studies that may have been missed during the electronic searches (secondary search). Final Search String: (pulses OR grains OR legumes) AND (fermentation OR “fermented foods” OR “solid-state fermentation”) AND (“glycemic index” OR “glycaemic response” OR antinutrients OR bioaccessibility OR “in vitro digestion” OR “starch digestibility” OR “resistant starch”).

### 2.3. Study Selection

Following the search, all identified citations were collated and uploaded into EndNote 20.0.1 (Clarivate Analytics, Philadelphia, PA, USA), and duplicates were removed. Subsequently, additional automated filtering procedures were performed in R Studio version 4.6.1 using the R statistical environment to support reproducible screening and corpus refinement, including the exclusion of review papers, books, conference abstracts, and studies related to silage or farm animal feeding. The complete R script used for these procedures is available in Supplementary Data S1. Then, two independent reviewers (J.A.C.B. and J.C.C.B.) screened titles and abstracts based on the pre-defined inclusion and exclusion criteria. Subsequently, the full texts of potentially relevant studies were retrieved and assessed in detail by the same reviewers. Any disagreements between reviewers were resolved through discussion or with the assistance of a third reviewer (C.H.P.). Reasons for the exclusion of full-text evidence sources that did not meet the eligibility criteria were recorded and will be reported in the scoping review using the PRISMA 2020 flowchart [19].

The eligibility criteria were structured to encompass studies focused on pulses/legumes bioprocessing and its associated nutritional and biological impacts (Table 1).

### 2.4. Data Charting Process

Data was extracted from the included studies independently by two reviewers using a standardized charting form developed by the research team. The AI-based tool NotebookLM (Google) was employed to assist in the initial data extraction process. To ensure the integrity and accuracy of the information, all AI-generated outputs were rigorously cross-checked and verified by both reviewers. The following data were extracted from each study: Study Details: Author(s), year of publication, country, and study design; Population (Matrix): Type of grain (legumes and pulse) and any applied pre-treatments; Concept (Fermentation): Microorganisms used, fermentation type (solid-state or submerged), process parameters (time and temperature), and intended application of the fermented matrix; Context (Results): Variation in antinutrient levels, changes in Glycemic Index or starch digestibility, protein digestibility, and the bioaccessibility of minerals or bioactive compounds; Outcomes: Main findings, study limitations, and identified research gaps.

### 2.5. Analysis and Presentation of Results

The data were presented in tabular and narrative form, in line with the objectives of the review. A descriptive analysis was performed to map knowledge gaps and the effectiveness of different types of fermentation on the glycemic and nutritional modulation of grains (pulses/legumes).

## 3. Results

### 3.1. Selection of Studies Results

**Figure 1:**
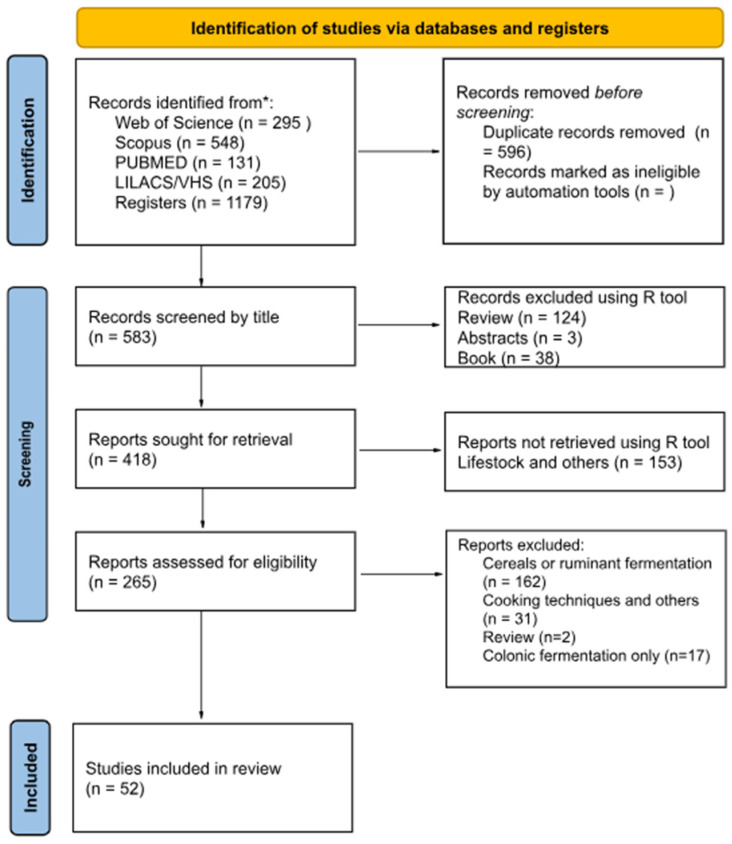
PRISMA 2020 Flow Diagram outlining the study selection process. The flowchart details the identification, screening, and inclusion phases of the scoping review.

The literature search conducted in April 2026 yielded a total of 1179 records identified across the selected databases: PubMed, Scopus, Web of Science, and LILACS/VHL. Following the initial identification, 596 duplicate records were identified and removed using EndNote software version 20 (Clarivate Analytics, Philadelphia, PA, USA) (Figure 1). To optimize the screening process, the remaining records were exported from EndNote in CSV format and processed in R Studio. Automated filtering scripts were executed to identify and exclude secondary literature (reviews), book chapters, and conference abstracts, resulting in the removal of 165 records. A subsequent computational filter was applied to exclude studies focused on silage production or livestock and poultry nutrition, leading to the exclusion of an additional 153 records. Finally, 265 records underwent manual screening of titles and abstracts by two independent reviewers to assess their adherence to the eligibility criteria. Following this comprehensive evaluation, 52 studies were selected for inclusion in the scoping review. The study selection process and flow are detailed in the PRISMA 2020 flowchart (Figure 1).

### 3.2. General Profile of the Included Studies

The 52 studies included in this review were published over a 10-year period, spanning from 2016 to 2026. These investigations encompass research conducted across multiple continents, including countries such as India, China, Italy, Spain, Nigeria, South Africa, Indonesia, Canada, Belgium, and Sweden. The selected studies applied fermentation processes to a wide diversity of grains, focusing primarily on traditional and underutilized endemic legumes (chickpea, lentil, pea, soybean, fava bean, red bean, cowpea, jack bean, lupin, velvet bean, and the wild seed Zamnè). Furthermore, these legumes were frequently combined with cereals and pseudocereals, namely sorghum, rice, wheat, and quinoa.

Data mapping revealed distinct experimental focuses within the included portfolio. Specifically, 14 publications directly evaluated alterations in protein digestibility via enzymatic assays, whereas 10 studies investigated starch fractions and the subsequent modulation of glycemic responses. Additionally, 11 documents quantified the bioaccessibility of essential minerals (Fe, Zn, and Ca) in gastrointestinal fluids following matrix biotransformation. Notably, most of the literature focused on phytochemical degradation; 32 articles simultaneously measured the reduction in antinutritional factors, such as phytic acid, condensed tannins, trypsin inhibitors, and raffinose family oligosaccharides (RFOs), and the concomitant increase in total phenolic compounds, flavonoids, and overall antioxidant capacity induced by fermentation. It is worth noting that several publications simultaneously investigated multiple nutritional and phytochemical attributes, resulting in overlapping categories.

## 4. Discussion

### 4.1. Fermentation of Pulses: Microorganisms, Process Dynamics, and Product Development

Pulse fermentation has emerged as a promising biotechnological strategy to enhance the nutritional, functional, and sensory value of legumes. This bioprocess enables the development of fermented plant-based beverages, protein ingredients, snacks, baked goods, and solid-state structured foods such as tempeh [1,2,20,21]. The selection of microorganisms, fermentation systems, and operational conditions directly dictates the resulting biochemical transformations. Consequently, these parameters modulate protein digestibility, antinutritional factor reduction, bioactive compound synthesis, technological stability, and the sensory profile of the final matrices [8,22,23]. Lactic acid bacteria (LAB) represent the most widely deployed microbial group, although yeasts and filamentous fungi are increasingly explored in submerged fermentation (SmF) and solid-state fermentation (SSF) to yield high-value plant-based ingredients [7,24].

The physiological nature of the selected microorganism governs the design of the fermentation system, dictating the physical state of the medium, incubation time, and acidification dynamics. LAB cultivations, predominantly using species such as *Lactiplantibacillus plantarum* and *Leuconostoc citreum*, are typically conducted in liquid media or highly hydrated matrices (SmF). These systems operate at 30–37 °C for relatively short periods (16–48 h), driving intensive organic acid production (mainly lactic and acetic acids via homofermentative and heterofermentative pathways) that shifts the matrix pH from approximately 6.5 down to 3.5–4.5 [8,25,26], as summarized in Table 2.

**Table 2:** Overview of microbial strains, operational conditions, and primary nutritional and phytochemical outcomes in pulse processing in submerged fermentation (SmF) systems.

Legume/Pulse	Pre-Treatment	Microorganism	Operational Conditions	Application	Reference
Pea	Aqueous formula of legume protein isolate	*Lactobacillus plantarum* strains	Starting from dissolved isolate concentrate for 24 h, bacterial inoculation (*L. plantarum*) or targeted hydrolase attack (Proteases/Phytases), supplemented with exogenous saturated volumes of inorganic minerals (FeC_l3_/ZnC_l2_).	Development of micronutrient carrier	Zhang et al. [27]
Chickpea (*Cicer arietinum* L.).	Aqueous solution with flours	*S. cerevisiae* LN7, *L. plantarum* 95, *P. lolii* B72, *L. diolivorans* 13–4 A, *L. mesenteroides* OM94.	30 °C for 24 h up to 48 h.	Plant-based beverage	Pazzanese et al. [8]
Cowpea, sorghum, and orange-fleshed sweet potato.	Flour	Mixed cultures (e.g., *Lactococcus lactis*, *Leuconostoc mesenteroides*, *L. pseudomesenteroides*).	Incubation at 35 °C for 48 h.	Functional food ingredient	Kewuyemi et al. [26].
Rice, lentils, wheat, barley, ragi, soybean, mung bean, cowpea, and chickpea.	Coarsely crushed grains.	*Lactobacillus plantarum* and *Lactobacillus* spp.	Incubated for 72 h at 37 °C with shaking at 200 rpm in the broth.	Composite food formula	Rajani et al. [28]
Faba bean and pea mixed with oat.	Mashed cooked seed purees	*Lactobacillus*, *Weissella*, *Pediococcus*, *Leuconostoc*, *Lactococcus* strains (MIX31/MIX33 mixtures).	24, 48, and 72 h operating at 30 °C or 37 °C; pH reduction to a range of 4.4 to 5.0.	Snack	Kahala et al. [29]
Pea (*Pisum sativum*).	Flour solution	*Lactobacillus plantarum* (ATCC 8014).	37 °C for 72 h.	Ingredient	Skalickova et al. [30]
Soybean (*Glycine max*).	Macerated mass—raw coagulated plant beverage.	Spontaneous Fermentation	24 to 96 h at a fixed temperature of 27 ± 2 °C during steepings.	Plant-based cheese (Soy-wara)	Adeyeye et al. [31]
Soybean, kidney bean, and mung bean.	Flour	Spontaneous Fermentation	72 h in a dark environment at room temperature, with a steeping ratio of 1:3 (*w*/*v*).	Functional ingredient	Nadhifa et al. [4]
Chickpea (*Cicer arietinum* L.).	Cooked puree	*Lentilactobacillus diolivorans* 13–4 A.	30 °C incubation for 48 h in diluted mixture; pH drop (from 6.23 to 4.33).	Baking ingredient	Chiacchio et al. [32]
Germinated brown rice and chickpea.	Homogenized mixture transformed into puree/beverage.	*Lactobacillus plantarum* BNCC194165.	48 h of incubation; 37 °C; initial pH 6.32 dropped to 3.33.	Functional plant-based beverage	Wu et al. [23]
Cowpea (*Vigna unguiculata*), Bira and Nakare cultivars.	Whole grains	Spontaneous Fermentation	72 h; 25 °C; final pH reached an average of 5.6; immersed at a 1:3 ratio in water.	Gluten-free savory crackers	Kanime et al. [33]
White rice, green/black mung beans, and chickpea (*O. sativa*, *V. radiata*, *P. mungo*, and *Cicer arietinum*).	Fluid batter	Spontaneous Fermentation	Mixtures incubated for 14 uninterrupted hours following process steps after an extra-long pre-soaking of 10 h.	Fermented dumplings	Kumari et al. [34]
Soybean (soy protein isolate—SPI).	Protein isolate powder converted into a gelled emulsion.	*Lactobacillus casei* 1.0038.	6 h of incubation; 37 °C; No pH adjustment.	Delivery vehicle (emulsion gel) for encapsulating lipophilic bioactive compounds	Han et al. [35]
Lentil (*Lens culinaris var. Castellana*).	Whole flour.	*Lactobacillus plantarum* CECT 748, along with the proteolytic enzyme Savinase 16 L.	15 h; 37 °C; pH artificially and constantly maintained alkaline at 8.5; vigorous shaking at 300 rpm.	Functional ingredient	Bautista-Expósito et al. [36]
Chickpea, mung bean, rice, wheat, pearl millet, black gram, and sorghum.	Wet batters	Spontaneous Fermentation	Germination: 16 h soaking, 48 h resting, room temp. Batter Fermentation: 10 h soaking, 14 h resting, room temp.	Preparations consumed in Indian diets/breakfasts	Khanam et al. [37]
White lima bean (*Phaseolus lunatus*).	Flour	Spontaneous Fermentation	0, 24, 48, 72, and 96 h; Room temperature.	Ingredient for bakery products	Ojo et al. [38]
Jack bean (*Canavalia ensiformis*).	Flour.	*Lactobacillus plantarum* IIA-1A5.	18 h at 37 °C; followed by 1 autoclaving cycle.	Ingredient for baked goods and functional products	Setiarto et al. [39]
Pea (*Pisum sativum*), Lentil (*Lens culinaris*), and Faba bean (*Vicia faba*).	Milled wheat and mashed grains immersed in water	*L. brevis*, *P. pentosaceus*, *L. fermentum*, *P. freudenreichii*, *L. plantarum*, *L. lactis*, and *L. mesenteroides*.	LAB: 2 days at 30 °C. LAB + PAB: 3 days at 30 °C anaerobically followed by 1 day at room temp aerobically; moisture adjusted between 10 and 19%.	Vegan yogurt	Kahala et al. [22]
Faba bean (*Vicia faba* L.)	Dehulled flour (DF) and protein-rich flour (PR)	*Leuconostoc citreum* TR116	48 h at 30 °C; initial pH ~6.4	Ingredient for wheat bread fortification	Hoehnel et al. [25]
Oat and Faba bean	Flour (oat) and concentrates (faba)	*Lc. paracasei*, *Leuc. citreum*, *L. brevis*, *L. namurensis*	20 h at 30 °C; final pH 4.1–4.3	Apple-enriched yogurt-style beverage	Viretto et al. [1]
Soybean	Soybean solution (milled and boiled grains)	*L. kefiranofaciens*, *L. kefiri*, *Kazachstania unispora*	36–48 h at 28 °C	Soy kefir (functional beverage)	Luo et al. [40]
Black chickpea	Flour	*Lactiplantibacillus plantarum* T0A10	24 h at 30 °C; Dough Yield (DY) 285	Enriched pasta	De Pasquale et al. [41]
Lentil, Bean, Chickpea, Pea	Gelatinized and raw flour	*L. plantarum* MRS1, *L. brevis* MRS4	24 h at 30 °C	Functional ingredients	De Pasquale et al. [42]
Yellow field pea (*Pisum sativum* L.).	Flour	*L. bulgaricus*, *S. thermophilus*, and *L. acidophilus*.	Incubation at 42 °C until pH reached 4.5 (biological acidification).	Ingredient for plant-based beverages.	Ma et al. [43]
Rice, Lentil, and Chickpea mix.	Flour mixture for yogurt-style snack.	*L. plantarum* DSM33326 and *L. brevis* DSM33325.	16 h at 30 °C; kinetic monitoring via Gompertz.	Vegan yogurt	Pontonio et al. [21]
Red Haricot Bean (*P. vulgaris* L.).	Whole grain fermented in brine.	Native LAB from the grain.	120 h at 25 °C; final pH reaching 3.8 to 5.2 depending on the solution.	Whole grain for consumption	Kitum et al. [44]
Cowpea and Green mung bean.	Aqueous extract	*Lactobacillus casei*.	Fermentation until pH 4.8 at 37 °C; optimization via RSM.	Functional synbiotic beverage	Chaturvedi et al. [45]
Faba bean (*Vicia faba* L.).	Flour in suspension.	*L. plantarum* VTT E-78076.	24 h at 25 °C in liquid suspension (1:3 flour/water).	Protein-dense ingredient for baking	Rosa-Sibakov et al. [46]
Pea (*Pisum sativum*)—sP residues.	Milled grain mixed with water (40:60 ratio).	*Lactiplantibacillus plantarum* ITM21B.	14 h at 37 °C; final pH recorded at 4.43.	Ingredient for wheat bread fortification	Di Biase et al. [47]

Conversely, filamentous fungi such as *Rhizopus oligosporus*, *Pleurotus ostreatus*, *Trametes versicolor*, and *Aspergillus* spp. are tailored for SSF (Table 3), where matrix moisture is rigidly maintained between 50% and 65% under moderate temperatures (25–30 °C) over prolonged cycles spanning 3 to 14 days [24]. Unlike LAB, fungal systems often display a gradual pH increase to alkaline levels (~7.2) during advanced stages due to progressive carbohydrate depletion and enhanced protein catabolism, which releases alkaline nitrogenous compounds such as ammonia [2].

These contrasting physiological behaviors reflect distinct ecological niches. While LAB thrive in high water activity (aw) environments, producing organic acids that rapidly colonize the matrix and inhibit competing microflora, filamentous fungi possess an obligate aerobic metabolism requiring highly porous matrices to facilitate oxygen transfer, hyphal expansion, and extracellular enzyme secretion. To optimize fungal SSF, initial matrix acidification via the incorporation of 3% to 6% citric acid has been successfully deployed [5]. This strategy immediately reduces the baseline pH to ~4.0, which maximizes the catalytic performance of acid-tolerant fungal hydrolases (e.g., phytases and alpha-galactosidases) while concurrently acting as a chelating agent to mitigate metallic ion inhibition.

The fermentation window acts as a critical threshold for nutritional and structural outcomes. Short LAB cycles (16–20 h) optimize antinutritional degradation and microbial stability without incurring excessive acidification [1]. For fungal SSF, extended windows (up to 14 days) support continuous mycelial growth and biomass accumulation, yielding superior antioxidant and phenolic releases [24]. However, excessive incubation (e.g., up to 120 h) may trigger fungal sporulation, leading to substrate browning and compromised protein digestibility [2].

The structural and compositional traits of target legumes further align them with specific technological applications. Dispersible matrices processed as flours or slurries (e.g., chickpea, pea, and lentil) are uniquely suited for LAB-driven SmF to produce plant-based beverages, yogurt-like snacks, and functional ingredients. In these colloidal systems, high hydration accelerates acidification, synthesizes exopolysaccharides, and refines rheological properties to improve colloidal stability and flavor profiles [1,23]. Concurrently, LAB fermentation effectively mitigates legume off-flavors; the metabolic transformation of volatile aldehydes (such as hexanal and nonanal, generated by endogenous lipoxygenases) reduces green and astringent notes, replacing them with desirable buttery and fruity metabolites like diacetyl and acetoin [32]. In contrast, whole or structurally resilient seeds (e.g., soybean, lupin, and *Zamnè*) are ideal substrates for fungal SSF. Here, the dense mycelial network physically integrates the cotyledons, synthesizing extracellular enzymes that partially degrade plant cell walls to transform tough textures into cohesive, structured foods [2,60]. This structural modification is particularly crucial for unconventional, highly resilient matrices like *Mucuna pruriens* and *Canavalia ensiformis*, which require severe thermal pre-treatments combined with fungal SSF to break down specialized toxic compounds like L-DOPA and concanavalin A [59].

Emerging avenues in pulse fermentation target niche clinical and industrial formulations. For instance, fungal fermentation of lentil and quinoa flours has been utilized to design soft texture-modified gels and bakery items tailored for elderly populations suffering from dysphagia. By correlating structural remodeling with decreased hardness and masticatory force, this approach addresses a critical gap in geriatric nutrition [6]. Conversely, when integrating fermented pulses into conventional wheat-based baking, technological challenges arise. The incorporation of fermented fava bean or chickpea flours alters the viscoelastic gluten network, reducing gas retention and specific loaf volume while accelerating staling [41,47]. Consequently, partial wheat substitution is typically restricted to thresholds below 20%, whereas non-aerated matrices (beverages, pastes, and yogurts) tolerate much higher pulse inclusion levels without structural failure [25].

Despite the abundance of bench-scale prototypes, understanding the metabolic behavior of microbial consortia under dynamic industrial conditions remains a significant bottleneck. Most available literature assesses static fermentations with constant parameters, ignoring the thermal gradients, moisture heterogeneity, and localized metabolite accumulation inherent to large-scale bioreactors.

During industrial scale-up, spatial and temporal environmental perturbations, such as mass transfer limitations, localized pH drops, dissolved oxygen gradients, and mechanical shear stress from large-scale agitation, may significantly alter the ecological equilibrium of the fermentation medium. In multi-strain consortia, these perturbations could disrupt the delicate balance between competitive and cooperative interactions among different strains. For instance, sub-optimal mixing might cause localized accumulations of lactic or acetic acids, creating hyper-acidic microenvironments that potentially depress the growth of acid-sensitive strains while favoring highly tolerant phenotypes. This shift from a synergistic co-culture to a competitive exclusion state can alter microbial population dynamics over time. Consequently, these metabolic shifts may impact the biochemical performance of the consortium, leading to variable synthesis rates of extracellular enzymes, such as phytases, proteases, and alpha-galactosidases. Ultimately, this enzymatic instability can result in a loss of nutritional consistency in the final plant-based ingredient, manifesting as potential batch-to-batch fluctuations in antinutritional factor degradation, protein digestibility values (IVPD), and the target glycemic profile. Therefore, bridging the gap between laboratory models and commercial production requires comprehensive future research focusing on industrial scale-up, predictive stability modeling of microbial kinetics under stress, and advanced process control strategies to suppress environmental gradients and safeguard batch-to-batch functional consistency.

### 4.2. Modulation of Protein Digestibility in Fermented Legumes

Enhancing protein digestibility is a primary strategy to improve the nutritional value and bioaccessibility of storage proteins in pulses destined for plant-based formulations. Fermentation drives this enhancement by structurally modifying vegetable storage proteins (such as vicilins and legumes), reducing antinutritional factors, and increasing matrix susceptibility to human proteolytic enzymes. Consequently, significant increases in in vitro protein digestibility (IVPD) are consistently reported across diverse legume fermentation systems (Table 4). For instance, lactic acid fermentation elevated the IVPD of red kidney bean from 29.04% to 80.97% [58], while *Lactiplantibacillus plantarum* driven bioprocesses increased black chickpea IVPD from approximately 80% to 91% [41]. Similar upward trends occur in yellow peas [43] and blended cereal-legume fermented beverages [1]. This phenomenon is driven by microbial proteases and peptidases that execute a partial pre-hydrolysis of storage proteins into low-molecular-weight peptides and free amino acids, alongside the simultaneous degradation of phytates, tannins, and trypsin inhibitors that typically impair gastrointestinal proteases [38,53]. Meanwhile it is worth observing that these variations could be influenced by microbial strain differences, substrate composition, fermentation time and processing conditions (e.g., temperature, pH). The specific processing conditions, including microbial strains, fermentation time, temperature, and pH for these studies, are detailed in Table 2 and Table 3.

Conversely, solid-state fermentation (SSF) driven by filamentous fungi can result in limited gains or localized reductions in digestible protein fractions, depending on matrix traits and post-fermentation processing. Drabo et al. [2] observed that the IVPD of *Zamnè* tempeh decreased from 76.9% in cooked seeds to 74.0% after 48 h, reaching 73.8% at 120 h of fermentation. Similarly, fava bean bars fermented with *Pleurotus ostreatus* exhibited a lower soluble protein fraction than their non-fermented counterparts [56]. These divergent outcomes suggest that specific fungal strains divert free amino acids and peptides toward mycelial biomass synthesis, altering the final protein architecture. Furthermore, post-fermentation thermal treatments, such as baking, can accelerate Maillard reactions between liberated peptides and reducing sugars, generating protein aggregates and melanoidins that physically restrict enzymatic docking.

From an industrial perspective, managing this kinetic trade-off requires strategic bioprocess optimization to maximize IVPD while preventing high-quality protein depletion by fungal biomass. First, establishing precise bioprocess termination thresholds by monitoring enzymatic markers (such as phytase vs. protease activity ratios) allows manufacturers to halt SSF immediately after anti-nutrient degradation and before advanced proteolytic consumption begins. Second, adjusting the initial carbon-to-nitrogen ratio of the substrate with easily assimilable carbohydrates can protect essential amino acids, diverting fungal metabolism away from protein catabolism. Finally, implementing multi-stage temperature and humidity controls, such as lowering incubation temperatures or reducing water activity (aw) in the late fermentation phase, can downregulate fungal vegetative growth, balancing macromolecular preservation with anti-nutrient elimination.

To map these structural alterations beyond conventional global chemical assays (e.g., OPA, TCA, or pH-stat methods), advanced proteomic and peptidomic platforms (LC-MS/MS) have been deployed. Wu et al. [23] demonstrated that gastrointestinal digestion of *Rhizopus oligosporus* fermented tempeh predominantly yielded short-chain peptides (8 to 11 amino acids), whereas unfermented soybean retained a higher proportion of long-chain fragments (>13 amino acids) corresponding to cleavage-resistant regions. This peptidomic mapping confirms that fungal metabolic activity exposes cryptic cleavage sites originally buried within the native protein structure, shifting the post-digestion profile toward easily assimilable fractions.

The baseline structural complexity of the legume matrix and upstream mechanical processing also modulate post-fermentation IVPD outcomes. While tempeh-type fermentation increases gray pea protein hydrolysis, protein coagulation methods (e.g., tofu production) yield even higher digestibility values [61]. This disparity highlights the role of the plant cell wall as a physical barrier against pancreatic protease diffusion. Consequently, combining intense maceration or fine milling with fermentation drives higher IVPD increases than fermenting intact, thermally treated whole seeds [50], as physical disintegration acts synergistically with microbial biochemistry to expose hidden polypeptide chains.

Despite extensive bench-scale mapping of initial bioaccessibility via static simulated gastrointestinal models like the INFOGEST protocol [41,56,58], empirical evidence regarding systemic bioavailability, transepithelial transport, and in vivo physiological impacts remains limited. Static artificial fluids simulate solubility and luminal peptide release but fail to capture complex physiological dynamics, including brush-border membrane peptide transport across enterocytes, competitive cellular uptake, and post-absorptive systemic metabolism. Gaps also persist regarding how fermentation-derived peptides interact with the human intestinal microbiota. Integrating complementary in vitro cell models (e.g., Caco-2 monolayers) and controlled clinical trials is essential to definitively establish the systemic nutritional functionality of fermented plant-based proteins.

### 4.3. Modulation of Starch Digestibility and Glycemic Index

Scientific literature demonstrates that controlled fermentation driven by specific microbial strains or homofermentative lactic acid bacteria (LAB) acts as a structural modulator capable of significantly increasing and retaining the resistant starch (RS) fraction within grain matrices (Table 5). For instance, solid-state fermentation (SSF) with *Pleurotus ostreatus* elevated the RS content of lentil flour from 66.5% to 76.2% [53]. Substantial increases have also been documented in sorghum (from 27.51 to 56.73 g/100 g) and cowpea matrices [26], as well as in black beans, where combining gelatinization with lactic fermentation drove an increase from 9.13% to 15.92% [42]. Biochemically, these microorganisms preferentially consume digestible starch fractions as metabolic energy sources, thereby concentrating the resistant polymer fractions. Concurrently, the accumulation of organic acids alters granular thermodynamics and promotes starch retrogradation, which recrystallizes amylose and amylopectin chains into highly ordered structures.

**Figure 2:**
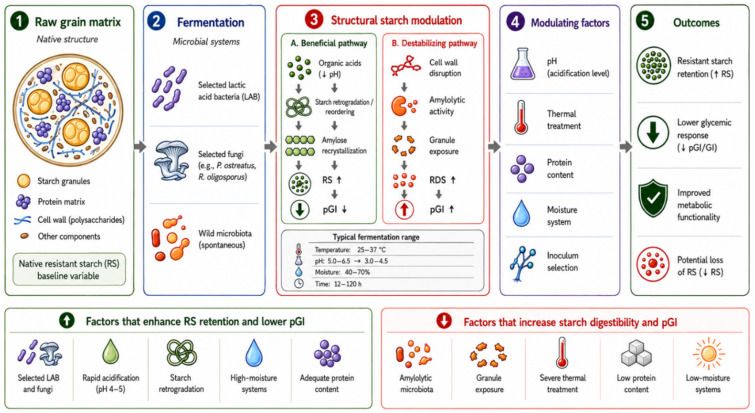
Mechanisms by which fermentation modulates starch structure, digestibility, and glycemic response. The scheme illustrates the main fermentation-dependent pathways affecting starch digestibility and glycemic response in grain and legume matrices. Arrows indicate the direction of the proposed effect or process flow; upward and downward arrows indicate an increase or decrease in the respective starch fraction or glycemic response. LAB, lactic acid bacteria; RS, resistant starch; RDS, rapidly digestible starch; pGI, predicted glycemic index; GI, glycemic index.

Consequently, this structural reorganization restricts starch hydrolysis rates and lowers the predictive glycemic index (pGI) and hydrolysis index (HI) of plant-based formulations. This hypoglycemic efficacy stems primarily from the microbial synthesis of organic acids (such as lactic and acetic acids), which drop the matrix pH to sub-optimal levels for human alpha-amylase activity while imposing steric hindrance that prevents enzymatic docking. For example, a legume-based yogurt-style snack developed with selected LAB exhibited a reduced pGI from an initial value of 60.6 to 53.4 [21], while fermented cereal–pulse beverages achieved a pGI drop from 68.3 to 53.4 [1]. Similarly, bread fortification with fermented pea ingredients yielded a low pGI of 50.24 [47], and lentil flours subjected to fungal SSF demonstrated a reduction in the maximum glycolysis rate from 34% to 24% [53]. Figure 2 summarizes the dualistic impact of fermentation on starch digestibility, highlighting the beneficial mechanisms associated with resistant starch retention and pGI reduction, as well as the destabilizing pathways that increase starch hydrolysis and glycemic response.

Conversely, divergent structural responses within the starch granule have been reported, revealing that fermentation does not inherently reduce rapidly digestible starch (RDS) and may even trigger severe declines in RS. In yellow field peas, unmodulated fermentation compromised the natural structural barriers of the seed, reducing the baseline RS from 340 to 201 g/kg [43]. Spontaneous fermentations driven by wild microflora in white lima beans induced a systematic decline in RS from 6.12% to ranges between 4.10% and 5.92%, shifting the postprandial glycemic response index from 57.11 to 62.82 over a 96 h cycle [38]. This structural degradation occurs when wild microflora, yeasts, or highly amylolytic strains aggressively degrade the plant cell wall to utilize structural polysaccharides as carbon sources, leaving the starch granules hyper-exposed to digestive enzymes. This destabilizing mechanism is exemplified by the use of *Saccharomyces cerevisiae* in multi-grain substrates, which exponentially increased the RDS fraction from 60.8% to 83.9% [7]. This underscores the necessity of using selected LAB strains that preserve the cellular skeleton and create chemical barriers via rapid acidification without destroying the starch architecture.

The intrinsic macronutrient balance of the raw flour further dictates the glycemic impact of the bioprocess, where protein concentration acts as a critical physicochemical variable during fermentation. Lactic fermentation increased the HI of low-protein, dehulled fava bean flour from 99.9% to 119.0% during wheat bread baking [25]. In sharp contrast, the same bioprocess significantly reduced the HI (from 133.4% to 114.9%) when deployed in a high-protein fava bean matrix containing 61% protein. In protein-dense matrices, the acidity generated by LAB induces extensive protein aggregation, denaturation, and folding. This structural rearrangement triggers a competition for available water, restricting local hydration and preventing the complete gelatinization of starch granules during baking, thereby preserving their crystalline organization against pancreatic amylase attack.

The efficacy of these enzymatic barriers is heavily dependent on the severity of downstream thermal treatments applied to the final food prototype. Fungal fermentation efficiently reduced starch hydrolysis in high-moisture gels but exerted no significant hypoglycemic effect when the same fermented flours were subjected to dry baking [6]. Similarly, combining fermentation with intensive pasteurization or autoclaving disrupted the native boundaries of yellow peas, causing digestible starch availability to surge from 88 to 258 g/kg [43]. Severe thermal processing induces a phase transition that collapses starch crystallinity (gelatinization), overriding the molecular barriers established by prior acidification. Conversely, in low-disruptive or high-moisture systems (such as gels), intact matrix polysaccharides and microbial exopolysaccharides can coat the remaining starch regions, establishing a physical coating that limits amylase accessibility.

Despite extensive bench-scale mapping of starch hydrolysis, extrapolating pGI values from in vitro models to in vivo human clinical, endocrine, and metabolic responses remains a significant bottleneck. Current evidence supporting low glycemic claims or high RS fractions relies heavily on static digestion models (such as the INFOGEST protocol), which estimate glycemic profiles solely by measuring continuous glucose release into a synthetic fluid over 180 min [21,47,53]. Isolated in vitro systems cannot replicate complex, interrelated physiological responses, such as the organic acid-mediated delay in gastric emptying, pancreatic insulin secretion dynamics, intestinal incretin responses, and colonic microbiome interactions. Consequently, while simulated digestion data are promising, randomized controlled human clinical trials are indispensable to confirm these metabolic outcomes and validate the therapeutic potential of fermented legumes against chronic metabolic disease.

### 4.4. Bioaccessibility and Bioavailability of Minerals in Fermented Legumes

The bioaccessibility and bioavailability of minerals in pulse-based products depend on the physicochemical interactions established between micronutrients and structural components of the plant matrix, primarily phytates, dietary fibers, and storage proteins. Phytic acid possesses a high chelating capacity at physiological pH, forming insoluble complexes with multivalent cations (Fe^3+^, Zn^2+^, and Ca^2+^) that drastically reduce their luminal solubility and subsequent intestinal absorption. During fermentation, the synergistic action of thermal pre-treatments, hydration, and microbial metabolism promotes a partial disruption of the plant cell wall, reducing chelating compounds and increasing the soluble mineral fraction within digestive fluids [2,7,49]. This dechelation process is driven by the production of organic acids that lower the medium pH, which deactivates the binding capacity of inositol hexaphosphate (IP_6_) and triggers progressive dephosphorylation via microbially secreted or endogenous seed phytases [3]. Consequently, a significant decline in phytate-to-mineral molar ratios (Phy:Fe and Phy:Zn) is achieved, releasing free ions into more soluble, bioaccessible forms [34,48].

Empirical evidence confirms this mineral mobilization across diverse fermentation systems (Table 6). Solid-state fermentation (SSF) driven by *Rhizopus* spp. severely degraded phytate levels in fava beans, peas, and *Zamnè* seeds, significantly improving zinc dialyzability and driving a sharp increase in cellular iron uptake, as validated by intracellular ferritin synthesis in human epithelial models [2,61]. Similarly, selenium-enriched soybean fermented with *Bacillus subtilis* natto displayed enhanced bioaccessibility of Fe, Mn, Cu, and Zn during simulated gastrointestinal transit [49]. Moreover, *Saccharomyces cerevisiae* drove a 64.5% reduction in the phytate core of multi-grain substrates, exponentially increasing the fraction of free mineral ions [7].

However, mineral bioaccessibility outcomes are not uniform across all matrices and elements, shifting according to specific process parameters and starting material structures. For instance, while lactic acid fermentation successfully expanded the bioaccessible fractions of Mn, Mg, Zn, and Fe in whole pea flour [30], it exerted limited or even negative impacts on zinc bioaccessibility when applied to pea protein concentrates, where isolated enzymatic hydrolysis proved far superior [27]. Furthermore, spontaneous fermentations in traditional formulations (e.g., *idli* and *dosa*) failed to improve copper mobilization and actively reduced bioaccessible manganese [34]. Fungal SSF with *Pleurotus ostreatus* in baked fava bean items also led to declines in calcium and iron bioaccessibility [56]. These discrepancies reveal that short incubation windows may prevent adequate phytase synthesis, or that microbial metabolites can promote the formation of novel, insoluble complexes between free minerals, structural fibers, and cross-linked peptides. Fungal strains generally exhibit high extracellular secretion of phytases and cellulases during prolonged SSF cycles, whereas LAB predominantly rely on matrix acidification to activate native seed hydrolases. Crucially, in protein-isolated matrices or systems subjected to post-fermentation dry baking, liberated minerals may undergo re-complexation with denatured proteins or residual fibrous fragments, compromising their final solubility [27,56].

To counteract these matrix limitations, pairing fermentation with emerging physical technologies has gained traction. Harahap et al. [3] evaluated the integration of ultrasound pre-treatments during maceration and cooking with varying fungal fermentation temperatures (30 °C and 36 °C) in tempeh production. The combination of dual ultrasound processing and a 36 °C incubation window triggered the highest release and bioaccessibility of calcium and iron during simulated digestion. This intensification is governed by acoustic cavitation, which physically disrupts the plant cell wall, increases matrix porosity, and exposes internal mineral-binding sites. This mechanical disruption acts synergistically with *Rhizopus* spp. proliferation, accelerating enzymatic docking and the subsequent solubilization of entrapped mineral networks.

Despite extensive bench-scale mapping of initial solubility, a distinct gap persists between in vitro luminal bioaccessibility and actual systemic bioavailability. While advanced frameworks have incorporated Caco-2/HT29-MTX cell co-cultures to monitor cellular absorption biomarkers [61], the vast majority of literature restricts its scope to quantifying solubilized ions in simulated gastric and intestinal fluids [2,30,49]. Luminal solubilization does not guarantee efficient transepithelial transport under complex physiological conditions. Furthermore, concurrent proteolysis generates short-chain peptides capable of chelating liberated metal ions, forming coordination complexes that can either facilitate or actively inhibit active and passive transport across the enterocyte brush-border membrane. Future research utilizing targeted proteomics, peptidomics, advanced biomimetic cell models, and randomized controlled clinical trials is imperative to decode the precise biological fate and net bioavailability of minerals from fermented plant-based matrices.

### 4.5. Bioaccessibility of Bioactive Compounds in Fermented Legumes

The bioaccessibility of bioactive compounds in fermented pulses is directly linked to structural modifications driven by microbial metabolism on the plant cell wall, which liberates bound phenolic compounds and flavonoids. Fermentation with lactic acid bacteria (LAB), yeasts, and filamentous fungi consistently increases total phenolic content (TPC) and antioxidant capacity, as measured by ABTS, DPPH, and FRAP assays, across various legume and cereal matrices [23,26,32,48]. This phytochemistry mobilization is governed by the microbial secretion of hydrolytic enzymes (such as beta-glucosidases, esterases, and cellulases) that degrade the polysaccharide framework of the plant cell wall and cleave conjugated polyphenols into free, highly reactive forms (Table 7). Crucially, enzymes like beta-glucosidase catalyze the bioconversion of glycosylated phytochemicals into aglycones, as exemplified by the conversion of daidzin and genistin into daidzein and genistein during kefir-driven soybean fermentation [40]. This deglycosylation reduces molecular weight and polarity, accelerating passive diffusion and transepithelial permeation across the intestinal epithelium [3,36].

Despite the elevated baseline of free phenolics post-fermentation, their subsequent stability and bioactivity during simulated gastrointestinal transit show structural divergences. Some frameworks indicate that gastrointestinal digestion acts synergistically with fermentation, promoting a progressive, continuous release of matrix-bound polyphenols that expands the bioaccessible fraction and cell-mediated radical scavenging capacity [36,50]. Conversely, other investigations reveal that while luminal digestion increases total solubilized polyphenols, the net antioxidant activity of these bioaccessible fractions can drop significantly after gastric and intestinal transit [23,53,56]. This loss of efficacy is tied to the chemical instability of specific fermentation-derived phenolics under shifting luminal pH levels, enzymatic degradation, and localized interactions with digestive proteins, which induce oxidation or form poorly reactive complexes.

This variability is tightly dictated by the structural complexity of the food matrix and the severity of downstream thermal processing applied to the fermented prototypes. In solid matrices subjected to intense dry heating, such as baked fava bean protein bars, a marked decline in bioaccessible antioxidant activity occurs [56]. This suppression is driven by Maillard reactions, where high thermal energy triggers the formation of high-molecular-weight melanoidins and complexes that entrap or oxidize phenolic structures, limiting their chemical availability. In contrast, liquid or low-disruption systems, such as soy-based beverages or blended brown rice and chickpea formulations, exhibit superior preservation of antioxidant properties and higher recovery of soluble phenolics post-digestion due to less severe thermal processing and minimal structural compaction [23,40].

To overcome these matrix limitations and move beyond descriptive in vitro solubility metrics, recent strategies have integrated emerging physical technologies with advanced biomimetic models [48,53]. Harahap et al. [3] paired acoustic cavitation via ultrasound pre-treatment with optimized fungal fermentation at 36 °C in tempeh production, achieving enhanced breakdown of the cell wall matrix and a subsequent surge in flavonoid bioaccessibility. Moving toward transepithelial transport modeling, Chiacchio et al. [32] deployed Caco-2 human epithelial cell monolayers to evaluate the active permeation of the bioaccessible fractions from fermented chickpea flours. This approach confirmed high cellular permeability for specific metabolites, such as pyrogallol, demonstrating how microbial biotransformation directly enhances net bioavailability.

A critical gap remains, however, regarding the biological fate of these bioaccessible fractions within the lower gastrointestinal tract, particularly their metabolic crosstalk with the human colonic microbiota. Most standard protocols restrict analyses exclusively to the upper gastrointestinal phases [36,53]. Addressing this limitation, Gumienna et al. [58] integrated a multi-stage system simulating colonic fermentation with human fecal microbiota, capturing dynamic shifts in polyphenol architecture and antioxidant profiles that peaked after 18 h of incubation. Non-absorbed polyphenols entering the colon undergo extensive microbial catabolism, generating low-molecular-weight secondary metabolites that exert local anti-inflammatory and antioxidant pressures while reshaping the microbial ecosystem. Consequently, robust validation of the health-promoting claims of fermented plant-based foods necessitates the routine implementation of integrated models that couple upper gastrointestinal digestion with colonic fermentation and host–microbiome interface analysis.

### 4.6. Phytochemical Antinutrients in Fermented Legumes

#### 4.6.1. Phytic Acid in Fermented Legumes

Scientific literature demonstrates that fermentative bioprocessing, whether driven by lactic acid bacteria (LAB), yeasts, or filamentous fungi, acts as an efficient mechanism to degrade phytic acid reserves and reduce antinutritional (Table 8). Solid-state fermentation (SSF) with *Rhizopus oligosporus* successfully decreased phytate contents in chickpea, pigeon pea, and soybean [48]. This reduction was also observed in fava bean flour when combining *R. oligosporus* and *Aspergillus oryzae* [20]. Similarly, selected strains of *Lactiplantibacillus plantarum* reduced phytic acid by up to 91.3% in wheat and 0.6% in cowpea [28], while probiotic *Saccharomyces cerevisiae* drove a 64.5% decrease in multi-grain substrates [7].

**Figure 3:**
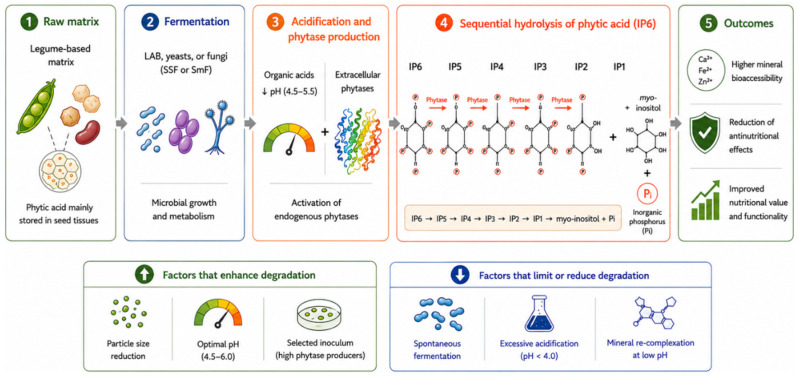
Mechanisms by which fermentation reduces phytic acid and improves mineral bioaccessibility. The scheme summarizes phytate degradation through acidification, phytase activation, extracellular phytase production, and sequential IP_6_ hydrolysis. LAB, lactic acid bacteria; SSF, solid-state fermentation; SmF, submerged fermentation; IP_6_, inositol hexakisphosphate; IP_5_–IP_1_, lower inositol phosphates; Pi, inorganic phosphate.

This degradative efficiency is governed by a dual enzymatic pathway. Initially, microbial metabolism synthesizes organic acids that lower the matrix pH, reaching sub-optimal to optimal ranges (typically 4.5–5.5) that activate endogenous seed phytases. Concurrently, starter cultures secrete high concentrations of extracellular phytases. This sequential dephosphorylation cleaves the phosphate rings of the inositol hexaphosphate (IP6) molecule, directly mitigating its mineral-chelating capacity. Consequently, fermentation establishes itself as an efficient non-thermal bioprocess to enhance mineral bioaccessibility in plant-based diets. Figure 3 summarizes the mechanistic pathways involved in phytic acid degradation.

Conversely, some investigations report limited degradation or even paradoxical increases in phytate contents, challenging the universality of this bioprocess. Unlike selected fungal inoculum, spontaneous fermentation reduced phytate levels in soybean and kidney bean by only 10% and 4%, respectively, underperforming when compared to germination, which achieved reductions of 26% and 22% [4]. More discrepant results occurred in velvet bean (*Mucuna pruriens*) flour, where phytic acid values rose from 1.50% to 4.06% after 24 h of *R. oligosporus* fermentation [59]. These variations stem from the complex interplay among inoculum profiles, intrinsic matrix properties, and shifting pH parameters. Spontaneous fermentations do not guarantee high phytase-producing strains. Furthermore, in recalcitrant legumes like *M. pruriens*, the metabolic stress induced by fungal proliferation can alter secondary biosynthetic pathways. This phenomenon generates lower-phosphorylated inositol intermediates and secondary compounds that cross-react during traditional colorimetric assays, leading to an overestimation of phytic acid content. Additionally, liberated mineral ions may undergo re-complexation with alternative structural components under lowered pH ranges, emphasizing that matching specific inoculum to individual botanical matrices is mandatory to avoid analytical artifacts and optimize degradation.

Upstream mechanical processing, specifically particle size reduction, also acts as a physical modulator that dictates phytase kinetics. Fungal SSF of lentils and quinoa using *Pleurotus ostreatus* achieved significantly higher phytate reduction rates in milled flours (89% and 90% drops) than in whole intact seeds (27% and 45% reductions, respectively) [24]. Fine milling disrupts the macro-architecture of the seed coat and cotyledon walls, substantially increasing the specific surface area. This physical disintegration allows fungal mycelia and extracellular phytases to penetrate the endosperm freely, accelerating molecular docking. In contrast, hydrolytic degradation in whole seeds occurs predominantly at the periphery due to slow internal enzyme diffusion, confirming that mechanical pre-treatments are as critical to antinutritional elimination as the metabolic capability of the selected strain.

Biochemical modulation of the fermentation medium further influences phytase performance, particularly through rapid shifts in acidity. In red haricot beans, salt-only solutions achieved higher phytate degradation (68.85%) than salt–sugar combinations (58.88%) [44]. Conversely, the incorporation of up to 6% citric acid into the matrix strongly restricted initial phytase activity during mushroom mycelial extrusion [5]. Fungal and bacterial phytases require precise optimal pH windows (typically 4.5–6.0) to maintain structural stability. When simple sugars are added, rapid microbial catabolism generates high concentrations of organic acids that drop the pH below critical thresholds (<4.0). This abrupt acidification causes the three-dimensional denaturation of phytase enzymes or promotes alternative insoluble interactions between proteins and inositol fractions, proving that carbohydrate concentrations and system buffering capacities dictate bioprocess efficiency.

Phytase enzymes, whether activated endogenously during soaking or biosynthesized by the microflora, remain the definitive biological vectors for phytic acid cleavage. Direct phytase treatments on fava bean demonstrated a cross-dependent relationship where higher enzyme loads correlated proportionally with sharp declines in refractory phytate fractions [46]. Similarly, intense colonization by basidiomycetes resulted in a 63% loss of phytic acid due to high mycelial phytase yields [5]. This underscores that targeted enzymatic hydrolysis remains mandatory to overcome the thermal stability of the phytate core.

Despite extensive bench-scale quantification of absolute phytate reduction, a major gap persists regarding the in vivo clinical bioavailability and systemic fate of residual inositol molecules within the gastrointestinal tract. Most standard protocols rely on the Wade reagent or traditional colorimetric precipitation to quantify global phytate loss, overlooking the exact chromatographic profile of degraded intermediates, such as inositol tri- and tetraphosphates (IP_3_ and IP_4_), present in the digestate [1,7,52]. Phytase hydrolysis is a stepwise process; therefore, various phosphorylated fragments can persist in the post-fermentation matrix. Traditional colorimetric assays are blind to the exact ionic status or the potential re-complexation kinetics of these intermediates when exposed to gastric acidity and proteolytic environments. Furthermore, because specific lower inositol derivatives exhibit antioxidant protective effects against free radicals under certain contexts, assuming that total reduction to zero is universally beneficial represents a simplistic approach. This limitation highlights the urgent need to transition toward high-resolution analytical platforms, such as liquid chromatography coupled with mass spectrometry (LC-MS/MS), to accurately track individual inositol fractions throughout the human digestive tract.

#### 4.6.2. Tannins in Fermented Legumes

Although tannins are widely recognized as potent polyphenol bioactive compounds due to their antioxidant capacity, they are systematically addressed in this subsection under an antinutritional scope. This specific analytical focus is selected because high-molecular-weight tannins establish strong cross-linking frameworks with dietary proteins and digestive enzymes, severely depressing protein digestibility and mineral bioaccessibility in raw legume matrices.

**Figure 4:**
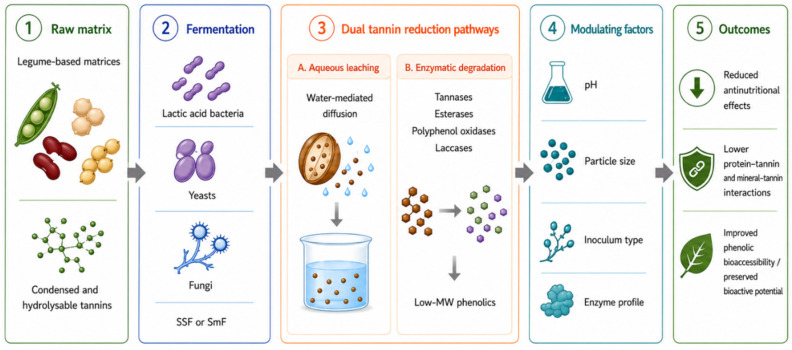
Mechanisms by which fermentation reduces tannins and modulates phenolic bioaccessibility. The scheme summarizes the main pathways involved in tannin reduction, including aqueous leaching and enzymatic degradation by microbial tannases, esterases, polyphenol oxidases, and laccases. LAB, lactic acid bacteria; SSF, solid-state fermentation; SmF, submerged fermentation; PPO, polyphenol oxidase; low-MW, low-molecular-weight.

The scientific literature demonstrates that fermentative bioprocessing, whether driven by lactic acid bacteria (LAB), yeasts, or filamentous fungi, acts as an efficient mechanism to degrade condensed and hydrolysable tannins within grain matrices. Significant declines in tannin concentrations are reported across multiple legume platforms. Spontaneous fermentation reduced tannin levels in fava bean flour from 35.29 mg/100 g to 19.05 mg/100 g [38], while *Lactiplantibacillus plantarum* driven processes decreased tannin contents in black chickpea snacks from 0.62 g/kg to 0.34 g/kg [41]. Furthermore, SSF using *Aspergillus oryzae* and *Rhizopus oligosporus* achieved near-complete elimination of condensed tannins in fava beans [20]. Marked reductions have also been confirmed in red haricot beans [44] and soy-wara [31]. This degradative efficiency is governed by a dual removal pathway: aqueous soaking facilitates the structural leaching of these water-soluble polyphenols out of the seed coat, while microbial cultures secrete specialized hydrolytic enzymes, principally tannases (tannin-acyl hydrolases) and polyphenol oxidases. These enzymes cleave the covalent ester bonds and depside linkages of high-molecular-weight tannin–protein polymers, transforming them into low-molecular-weight, inert subunits like glucose and gallic acid, which mitigates astringency and improves protein digestibility. Figure 4 summarizes the dual pathways governing tannin reduction during fermentation, highlighting the combined effects of aqueous leaching, microbial tannase activity, polyphenol oxidation, and process-dependent modulation of tannin degradation efficiency.

Conversely, some investigations report limited degradation or even paradoxical increases in tannin contents, challenging the universality of this bioprocess. Unlike selected fungal inoculum, SSF driven by *R. oligosporus* increased tannin levels by 8%, 10%, and 43% in desi chickpea, pigeon pea, and soybean, respectively [48]. Similarly, tannin values rose from 0.38% to 0.50% in velvet bean (*Mucuna pruriens*) flour after 24 h of bioprocessing [59]. Furthermore, LAB starter cultures exerted a neutral, statistically insignificant effect on the condensed tannins of lentils and fava beans across the majority of evaluated consortia [22]. These discrepancies stem from the structural complexity of polyphenols within specific plant cell walls. Fungal enzymatic shock can induce partial cleavage of highly polymerized, insoluble proanthocyanidins, liberating smaller, soluble tannin fragments that cross-react with colorimetric reagents, thereby causing an artificial inflation of quantified values. Additionally, the failure of certain bacterial strains indicates that tannase-encoding genes are not ubiquitous, depending strictly on strain-specific metabolic traits and matrix recalcitrance.

The biochemical modulation of the fermentation medium further influences tannase performance, particularly through rapid shifts in acidity. In red haricot beans, submerged salt-only fermentation (SOF) achieved higher tannin degradation (73.19%) than salt–sugar fermentation (SSF), which reached a 64.70% reduction [44]. Fungal and bacterial tannases require precise optimal pH windows (typically near 6.5) to maintain structural stability. When simple sugars are incorporated, rapid microbial catabolism triggers a massive bacterial growth phase that drops the medium pH to 3.88. This abrupt acidification induces the three-dimensional denaturation or early inhibition of the tannase enzyme. Conversely, the salt-only formulation maintains a moderate environment (pH 5.26), prolonging the catalytic peak necessary to hydrolyze the tannin polymer, which proves that carbohydrate formulation and buffering capacity dictate in situ enzymatic efficiency.

Upstream mechanical processing and the growth morphology of the fermenting agent (bacteria versus filamentous fungi) also act as physical modulators that dictate tannin kinetics. Intact jack bean seeds subjected to unmodulated thermal processes exhibited negligible tannin decreases, shifting only from 0.35 g/100 g to 0.34 g/100 g [57]. In sharp contrast, applying matrix flours to the intensive colonization of white-rot fungi (*Trametes versicolor*) or *R. oligosporus* in solid-state cultures achieved a 90–100% degradation of the tannin framework [5,20]. Fungal mycelia possess a mechanical advantage, physically penetrating the pericarps and fibrous cell walls to excrete robust hydrolase mixtures directly onto insoluble tannin-binding sites. In submerged LAB or spontaneous systems without mechanical cellular disruption, bacterial cells remain restricted to external surfaces, failing to access or degrade tannin–protein complexes embedded deep within the endosperm.

Despite extensive bench-scale quantification of total tannin reduction, a major gap persists regarding the in vivo clinical bioavailability and systemic fate of degraded oligomers within the human gastrointestinal tract. Most standard protocols rely exclusively on traditional colorimetric extractions and spectrophotometric assays, such as the Folin–Denis or vanillin-HCl methods, to report a simple percentage of global tannin loss in freeze-dried products [26,31,38]. These colorimetric screening tools provide binary data (presence versus absence) but remain blind to the exact molecular architecture of newly biotransformed fragments, such as free catechins and short chain proanthocyanidins. Modern food science lacks systematic tracking via liquid chromatography coupled with mass spectrometry (LC-MS/MS) to determine whether these fermentation-derived residues completely lose their mineral-chelating capacity or acquire novel immunomodulatory and prebiotic functionalities beneficial to the host microflora. This highlights the urgent need to transition toward high-resolution degradomic assays linked to systemic physiological trials.

#### 4.6.3. Oligosaccharides and Protease Inhibitors in Fermented Legumes

**Figure 5:**
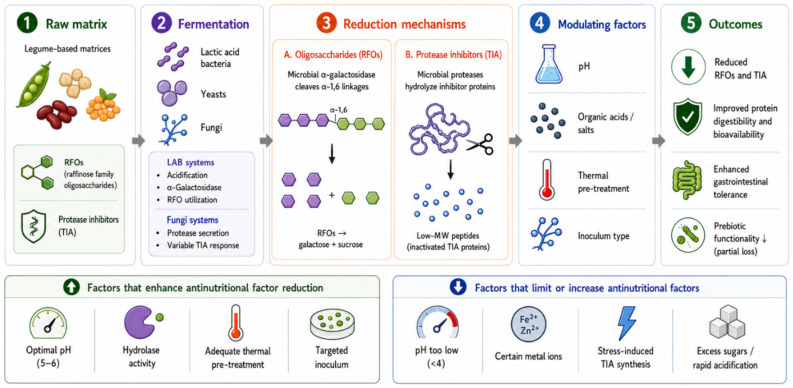
Mechanisms by which fermentation reduces raffinose family oligosaccharides and trypsin inhibitor activity. The scheme summarizes the main pathways involved in the reduction in raffinose family oligosaccharides and trypsin inhibitor activity, including α-galactosidase-mediated hydrolysis of α-1,6 linkages and protease-mediated degradation of inhibitor proteins. LAB, lactic acid bacteria; RFOs, raffinose family oligosaccharides; TIA, trypsin inhibitor activity; low-MW, low-molecular-weight.

Scientific literature demonstrates that fermentative bioprocessing represents a highly effective approach to mitigate flatus-inducing galacto-oligosaccharides and hydrolyze protease inhibitors, thereby enhancing gastrointestinal tolerance and protein bioavailability. Fermentation driven by selected lactic acid bacteria (LAB) and specific fungal strains actively eliminates raffinose family oligosaccharides (RFOs). Drastic reductions exceeding 90–95% in raffinose, stachyose, and verbascose contents have been reported in peas, fava beans, and haricot beans [22,44]. Concurrently, a systematic degradation of trypsin inhibitor activity (TIA) occurs during the fermentation of yellow field peas [43], sweet lupin [51], and black chickpea flours [41]. This consensus is anchored in microbial enzymology. The degradation of RFOs is driven by the microbial secretion of alpha-galactosidase, an enzyme that cleaves the structural alpha-1,6-glycosidic linkages that remain indigestible to humans due to the lack of this enzymatic activity in the upper gastrointestinal tract. In parallel, trypsin inhibitors are inactivated via extensive proteolysis by microbially secreted endopeptidases, alongside structural leaching into the aqueous medium during upstream soaking steps. SDS-PAGE profiling has confirmed that the collapse of TIA correlates with the enzymatic cleavage of storage proteins into low-molecular-weight polypeptide fragments (<16 kDa) [43]. Figure 5 summarizes the biochemical and physicochemical mechanisms governing these pathways.

Conversely, critical discrepancies exist regarding the universality of this antinutritional degradation, revealing that specific microorganisms fail to hydrolyze certain oligosaccharides or may even synthesize protease inhibitors. Unlike LAB-driven systems, SSF with *Rhizopus oligosporus* left raffinose and stachyose concentrations unaltered in fava bean flour [20], while *Pleurotus eryngii* failed to significantly modify RFO baselines in red kidney bean powder [5]. Notably, significant post-fermentation increases in TIA have been documented in soybean, common bean, pigeon pea, and fava bean. In these matrices, inhibitor activities surged from a non-detectable baseline to threshold levels of 4.22 TIU/mg following SSF with *R. oligosporus* or *Aspergillus oryzae* [20,48]. This RFO persistence stems from substrate-enzyme specificity, as certain fungal strains lack the specific alpha-galactosidases required to cleave stachyose, preferentially consuming alternative carbon sources. Meanwhile, the paradoxical increase in TIA occurs because filamentous fungi actively synthesize protease inhibitors as an evolutionary defense mechanism against environmental stressors during the colonization of raw or minimally processed solid substrates. This demonstrates that antinutritional elimination is dictated by the transcriptomic profile of the chosen inoculum.

The targeted physicochemical regulation of the fermentation medium, specifically using organic acids or salt formulations to modulate hydrolase efficiency, remains an effective strategy to optimize antinutritional breakdown. The incorporation of 3% citric acid into the matrix sharply intensified RFO degradation by *Trametes versicolor* [5]. Similarly, submerged salt-only fermentation (SOF) achieved higher RFO and antinutrient losses in red haricot beans than mixed salt–sugar fermentation (SSF) [44]. Mechanistically, microbial alpha-galactosidases are frequently inhibited by specific metallic ions, which are sequestered by the chelating action of citric acid, thereby restoring enzyme catalytic freedom. This demonstrates that carbohydrate formulation dictates system buffering capacity, preserving the optimal catalytic window (pH 5.0–6.0) required to sustain structural stability.

The structural variance in final TIA and RFO profiles is further driven by the synergistic interaction between upstream thermal severity and the biological phylum of the fermenting agent (Fungi versus Bacteria). Complete eradication of trypsin inhibitors is typically achieved when fermentation is paired with severe thermal pre-treatments, such as boiling velvet bean for 80 min [59] or pressure-cooking kidney bean powder [5]. Because trypsin inhibitors are thermolabile proteins, autoclaving or prolonged boiling induces irreversible denaturation prior to microbial inoculation. When raw or minimally heated seeds are used, the native inhibitor networks remain intact. Under these conditions, aggressive filamentous fungi trigger stress-induced cellular pathways that synthesize exogenous inhibitors. Additionally, LAB possess a specialized saccharolytic metabolism tailored for the direct consumption of RFOs to sustain cell viability [1], rendering them inherently more efficient at oligosaccharide elimination than wood-decay fungi focused primarily on structural fiber breakdown.

Despite extensive in vitro mapping, a major dichotomy exists regarding the clinical impacts of these bioprocesses on the human intestinal microbiota. Standard literature operates under the reductionist premise that the absolute elimination of RFOs and TIA is the sole objective required to optimize pulse functionality [1,5,22]. This perspective overlooks the fact that residual RFOs and their hydrolysis intermediates (such as melibiose) exert recognized prebiotic functions in vivo, selectively stimulating the proliferation of *Bifidobacterium* and the subsequent synthesis of beneficial short-chain fatty acids (SCFAs) in the colon. Achieving 100% RFO eradication resolves gastrointestinal discomfort but deprives the legume matrix of its core prebiotic value. Furthermore, the synthesis of trypsin inhibitors by *R. oligosporus* raises unanswered physiological questions regarding whether these fungus-derived proteins survive gastric pepsin digestion, if they induce the same nutritional deficits as native plant inhibitors, and how they modulate host immune responses. Resolving these bottlenecks requires an immediate transition toward randomized controlled clinical trials coupled with human fecal microbiome sequencing to validate the metabolic safety of fungal-mycelium-engineered foods.

### 4.7. Systematic Evaluation of Experimental Inconsistencies and Methodological Gaps

The synthesis of the published literature reveals that fermentative bioprocessing does not exert a unidirectional effect on legume matrices. Instead, the severe discrepancies and paradoxical outcomes reported across different studies, such as localized drops in protein digestibility, mineral re-complexation, or unexpected spikes in the predictive glycemic index, stem from a multi-variable matrix of technological and biological factors. To provide forward-looking guidance for functional food design, Table 9 systematically reorganizes these cores influencing variables, isolating the reasons behind conflicting experimental behaviors and mapping the critical transition from in vitro models to in vivo scalability.

## 5. Conclusions and Future Perspectives

This scoping review confirms that grain and legume fermentation represents a highly efficient biotechnological platform for the nutritional, rheological, and kinetic reconfiguration of whole seeds, isolates, and agro-industrial by-products. The success of this bioprocess hinges on a precise coordination of mechanisms dictated by the biological phylum of the chosen inoculum. Matrix acidification driven by homofermentative lactic acid bacteria (LAB) and the mechanical penetration of fungal hyphae during SSF act synergistically to modulate grain microstructure and cleave complex antinutritional frameworks.

The expression and extracellular secretion of specific microbial enzymes, principally phytases, tannases (tannin-acyl hydrolases), polyphenol oxidases, and alpha-galactosidases, serve as the primary drivers to systematically dismantle plant chemical defenses. This enzymatic activity degrades phytic acid and condensed tannins while hydrolyzing non-digestible raffinose family oligosaccharides (RFOs). Concurrently, microbial proteases execute a targeted pre-hydrolysis of storage proteins (such as vicilins and legumes), reducing them into short chain neopeptides (predominantly 8 to 11 amino acids) and free amino acids. This synergistic breakdown drives substantial increases in vitro protein digestibility (IVPD), which reaches thresholds up to 80.97%, and promotes the structural solubilization of divalent and trivalent cations, thereby enhancing the dialyzability and intestinal bioaccessibility of iron, zinc, calcium, and magnesium. Additionally, microbial beta-glucosidases hydrolyze sugar moieties, converting conjugated glycosides into highly reactive free aglycones.

Furthermore, the high-titer synthesis of organic acids and microbial exopolysaccharides modulates carbohydrate kinetics through a dual pathway: lowering the matrix pH below the optimal thresholds for human alpha-amylase activity and inducing thermodynamic modifications that promote molecular retrogradation. This structural rearrangement recrystallizes amylose and amylopectin chains, significantly increasing the retention of resistant starch (RS) and slowly digestible starch (SDS) fractions at the expense of rapidly digestible starch (RDS). Consequently, the hydrolysis index (HI) is suppressed, maintaining the predictive glycemic index (pGI) within low thresholds. This structural softening can be tailored to design soft-textured gels for clinical geriatric formulations addressing dysphagia, while the microbial reduction of volatile aldehydes eliminates astringent off-flavors (such as hexanal and nonanal), ensuring palatable profiles for commercial plant-based dairy alternatives.

However, these nutritional and kinetic benefits are neither universal nor linear. As synthesized in this review, the structural and biochemical trajectory of fermented grains and legumes is strictly dictated by the core quadrant of parameters: substrate macronutrient distribution, initial processing moisture, precise pH optimal windows, and the severity of upstream thermal or mechanical pretreatments. Severe dry baking or unmodulated spontaneous flora can completely reverse the targeted functional outcomes, collapsing starch crystallinity and driving nutrient re-complexation. Intensive thermal treatments, such as dry baking, pasteurization, or autoclaving, induce an irreversible collapse of starch crystallinity, causing complete gelatinization that overrides the protective chemical barriers established during prior fermentation. This molecular disruption liberates the starch core, accelerating digestible starch availability and elevating postprandial glycemic responses. Similarly, unmodulated spontaneous fermentations driven by wild microflora lack high-performance, phytase-specific strains and excessively disrupt the cell wall architecture for energy consumption. This carbohydrate depletion forces the catabolism of structural membranes and proteins, generating ammonia spikes that shift the pH toward alkaline levels (~7.2), which triggers fungal sporulation, sensory deterioration, and RS degradation. Crucially, uncontrolled or extended fermentation cycles, especially those relying on wild microflora or unverified filamentous fungi, pose serious food safety risks, including the potential accumulation of toxic compounds such as biogenic amines (e.g., histamine, putrescine) via amino acid decarboxylation, or dangerous mycotoxins. Without strict strain purification and rigorous process controls, these biochemical hazards can severely compromise the safety profile of the final plant-based ingredient. Moreover, in isolated protein concentrates or solid systems lacking whole-grain structures, liberated minerals and synthesized polyphenols may undergo re-complexation with denatured proteins or Maillard-derived melanoidins, trapping bioactive compounds and compromising cellular antioxidant capacities.

Future avenues in this field necessitate a definitive transition from static bench-scale protocols to advanced, targeted research directions, which are systematically categorized into four strategic pillars:

- Strain Screening and Compound Starter Optimization: Future research must move beyond random selections and perform rigorous transcriptomic and metabolic profiling to match specific microbial strains to individual botanical varieties. Optimization should focus on developing defined multi-strain compound cultures (LAB, yeasts, and fungi) capable of targeting multiple anti-nutrients simultaneously without compromising the structural integrity of the grain proteins.
- Combined Physical Pretreatment and Synergistic Fermentation: Investigative efforts should prioritize the integration of emerging non-thermal processing technologies, such as acoustic ultrasound cavitation, high-pressure processing, or controlled germination, prior to inoculation. This mechanical disruption acts synergistically with microbial biochemistry to increase matrix porosity, accelerate enzymatic docking, and enhance the baseline release of bound bioactive phenolics and minerals.
- Integrated Multistage Digestion, Cell Assays, and Human Clinical Trials: To validate true biological functionality, investigations must shift from binary colorimetric assays toward high-resolution analytical platforms (LC-MS/MS) coupled with multi-stage in vitro colonic fermenters to track individual inositol fractions (IP3 and IP4) and phenolic catabolism by the human microbiota. This framework must be integrated with Caco-2/HT29-MTX co-cultures and randomized, controlled human clinical trials, which represent the only path to verify whether fermentation-derived peptides resist pepsin degradation and successfully translate into transepithelial transport, endocrine metabolic safety, and glycemic control in living hosts.
- Industrial Scale-Up Parameter Exploration: Future studies must explore large-scale bioreactor mechanics, modeling dynamic operational parameters such as mass transfer limitations, moisture heterogeneity, localized pH gradients, and thermal fluctuations. Addressing these industrial engineering barriers is mandatory to suppress environmental stress gradients, prevent toxic metabolic drift (such as biogenic amine or mycotoxin accumulation), and safeguard batch-to-batch functional and sensory consistency.

Finally, because current empirical evidence remains restricted to bench-scale simulations, it must be explicitly stated that no clinical or therapeutic recommendations can be established at this stage; randomized human intervention trials are a mandatory ethical prerequisite to validate any health-promoting claims and ensure consumer safety before these matrices are recommended for clinical applications. Also, this review scope did not standardized data regarding the sensory properties and consumer acceptance of the developed food prototypes, which represents a critical barrier to their industrial translation.

## Figures and Tables

**Table 1:** Eligibility Criteria.

Category	Inclusion Criteria	Exclusion Criteria
Study Design	Experimental in vitro studies (gastrointestinal digestion simulation), in vivo studies (animal or human models for glycemic index), and chemical characterization studies.	Literature reviews, editorials, and conference abstracts.
Intervention	Clearly described grain (pulses) fermentation process (lactic acid bacteria, yeasts, or fungi) including a comparison between unfermented and fermented matrices.	Studies using grains solely as a substrate for purified enzyme production without evaluating the food matrix. Studies using cereals or pseudocereals.
Outcomes	Must report at least one data point on: glycemic index, starch digestibility, antinutrient quantification, or nutrient/bioactive compound bioaccessibility percentage.	Studies assessing only sensory profiles or basic physicochemical parameters (e.g., pH, acidity) without biological or nutritional outcomes.
Context	Studies focused on human nutrition and food science.	Studies evaluating grain fermentation for livestock, poultry, or ruminant feed.

**Table 3:** Overview of microbial strains, operational conditions, and primary nutritional and phytochemical outcomes in pulse processing in semi solid fermentation (SSF) systems.

Legume/Pulse	Pre-Treatment	Microorganism	Operational Conditions	Application	Reference
Chickpea (kabuli and desi), pigeon pea, and soybean.	Whole grains	*Rhizopus oligosporus* MTCC 556.	Incubation for 52 h at 34 °C; no exact initial pH or moisture recorded in excerpts.	Ingredient for functional foods	Toor et al. [48]
Soybean fortified with selenium nanoparticles.	Autoclaved whole seeds	*Bacillus subtilis* subsp. *natto*	Autoclaved beans incubated under typical natto production conditions.	Natto	Zhao et al. [49]
Rice, lentils, and soybean (3:1:1 multi-grain blend).	Whole cooked complex in formulated mixed medium	*Saccharomyces cerevisiae* AKP1	Incubated for 4 days at 30 °C; medium pH dropped from 6.26 to 4.56, while aqueous levels (moisture) decreased.	Production of accessible nutritious functional food base	Banik et al. [7]
Chickpea, faba bean, lentil, pea, and kidney bean.	Flour, or cracked/whole grain.	*Lactobacillus plantarum* ATCC 8014	Incubation for 48 h at 37 °C (pH declined from 5.9–6.3 to 3.9–4.5 levels); mixtures contained balanced hydration rates (e.g., 1:1 *w*/*v*).	Bioactive functional food ingredients	Di Stefano et al. [50]
Lupin (*Lupinus mutabilis Sweet*) containing INIAP and Criollo varieties.	Dehulled and crushed grains	*Rhizopus oligosporus* NRRL2710	28 °C for 4 days in a restricted environment to 50% bran moisture.	Ingredient	Villacrés et al. [51]
Pigeon pea (*Cajanus cajan*).	Milled fractions of germinated grains	Spontaneous Fermentation	24, 48, and 72 h at room temperature 30 °C; pH dropped to the 5.30 range.	Baking ingredient	Sobowale et al. [52]
Zamnè (*Senegalia macrostachya* seeds).	Pre-cooked whole seeds	*Rhizopus oryzae*	Incubation from 0 to 120 h (48 h for fresh product); 30 °C; 70% relative humidity; initial pH 5.6 raised to 7.2.	Tempeh and ingredients for infant supplements	Drabo et al. [2]
Lentil, chickpea, and soybean.	Autoclaved whole seeds	*Pleurotus ostreatus*	Up to 14 days; temperature 28 °C; moisture 65%. Initial pH of 6.64, 6.74, and 6.50 for lentil, chickpea, and soybean (all reduced).	Plant-based ingredient	Noori et al. [9]
Lentil (*Lens culinaris*).	Whole grains	*Pleurotus ostreatus*	Incubation in a static chamber at 28 °C for 14 days.	Ingredient for low glycemic index formulations	Asensio-Grau et al. [53]
Faba bean (*Vicia faba* L.).	Whole grains	*Aspergillus oryzae* and *Rhizopus oligosporus*	A. oryzae: 30 °C/72 h. *R. oligosporus*: 28 °C/48 h. Humidification 1:2 *v*/*v* (pH 4.5–5.5 using vinegar for *R. oligosporus*).	Protein ingredient for plant-based foods	Gautheron et al. [20]
Lentil (*Lens culinaris*) and Quinoa (*Chenopodium quinoa*).	Whole grains	*Pleurotus ostreatus*	Initial moisture adjusted (65%), followed by 28 °C for 14 days.	Protein ingredient for plant-based foods	Badia-Olmos et al. [54]
Lentil (*Lens culinaris*), Pardina and Castellana varieties.	Thermally processed grains	*Pleurotus ostreatus*	14 days of incubation; temperature of 28 °C; hydrated matrix moisture at 65%.	Ingredient	Sánchez-García et al. [55]
Lupin (*Lupinus mutabilis*).	Whole grains	*Rhizopus oligosporus* ATCC NRRL2710	Grain humidified to reach 50% moisture before inoculation; 4-day (96 h) fermentation at 28 °C.	Baking ingredient	Villacrés et al. [51]
Faba bean (*Vicia faba* L.).	Compact dough (mixed cereal and food seed bar)	*Pleurotus ostreatus* CECT20311	Initial water soaking 16 h; moisture adjusted to 55%, SSF for mycelial development.	Snack formulation and diet bars	Khvostenko et al. [56]
Jack bean (*Canavalia ensiformis*).	Whole dehulled grains	Spontaneous Fermentation	70 to 72 h; room temperature 25–28 °C; wrapped in cellophane bags.	Ingredient	Arise et al. [57]
Red kidney bean (*Phaseolus vulgaris* var. Red Kidney).	Milled grain dough combined with aquafaba in wafer-style formulation	*Lactobacillus plantarum*	20 h of incubation; temperature of 37 °C; moisture adjusted to ~50%.	Healthy snacks/vegetable cookies	Gumienna et al. [58]
Quinoa and Lentil.	Flour	*Pleurotus ostreatus*	Solid state; drying at 70 °C.	Gels and breads for older adults	Gomez-Gomez et al. [6]
Lentil and Quinoa.	Whole grains and Flour	*Pleurotus ostreatus*	14 days at 28 °C; 65% moisture.	Ingredients	Sanchez-Garcia et al. [24]
Velvet bean (*Mucuna pruriens*).	Cooked dehulled grains	*Rhizopus oligosporus*	24 to 72 h at 29 °C; pH adjusted with vinegar.	Ingredient for food fortification	Ezegbe et al. [59]
Common bean (Kidney).	Flour	*P. eryngii* and *T. versicolor*	3 to 5 days at 28 °C; addition of citric acid (0–6%) for pH control.	Bean ingredient for plant-based foods	Li et al. [5]
Jack bean (*Canavalia ensiformis*).	Cooked grain	*Rhizopus oligosporus*.	48 h at 30 °C.	Ingredient	Purwandari et al. [60]
Soybean (*Glycine max*).	Whole grain	*Rhizopus oligosporus*	48 h at 30/36 °C.	Functional tempeh	Harahap et al. [3]
Soybean (*Glycine max*).	Defatted and milled grains	*Rhizopus oligosporus*	Standard commercial process (includes hydration and cooking) monitored by in vitro proteomics.	Tempeh—Nutritionally dense meat substitute	Wu et al. [23]

**Table 4:** Effects of fermentation on pulse protein digestibility and proposed mechanisms for the observed changes.

Change in Grain Protein Digestibility	Proposed Mechanism for the Change in Protein Digestibility	Reference
Tempeh-style fungal fermentation increased the average degree of protein hydrolysis (DH) to 47%, compared to 37% measured in exclusively cooked legumes. A statistically significant improvement in digestibility, relative to the cooked control, was specifically attested for gray pea tempeh.	The mechanism relies on the activity of proteolytic enzymes actively secreted by the fungus (e.g., *Rhizopus oligosporus*) during solid-state fermentation. This activity induces prior hydrolysis of large storage proteins, releasing smaller peptides and free amino acids, making the structure highly accessible to human gastrointestinal proteases.	Auer et al. [61]
An increase in the extent of protein degradation occurred. In yellow pea flour, fermentation raised the pre-digestion degree of hydrolysis (DH) from 9.91 (raw) to 14.00 meqv Ser-NH2/g. After exposure to simulated gastrointestinal fluids, the sample continued to show severe hydrolysis, confirming the high susceptibility of protein bands to digestive enzymes.	The process is primarily mediated by the acidification of the medium, which activates the endogenous proteinases of the seed. Subsequently, the strong intrinsic proteolytic activity of the *Lactobacillus plantarum* inoculum cleaves complex native proteins into low molecular weight peptide fractions (below 10 kDa).	Di Stefano et al. [50]
There was a significant surge in the concentration of digested proteins and the release of free amino acid chains. Following digestion, fermented pea flours exhibited a marked increase in specific amino acid fractions (such as Cysteine, Methionine, and Glutamine) and resulted in a significantly higher concentration of accessible soluble protein in the digesta when compared to the raw control.	Bioconversion by the bacterium *Lactobacillus plantarum* acts by disrupting and consuming the polysaccharide wrapping matrix of the flour. This architectural breakdown extinguishes the physical barriers blocking the granule, granting free access for the catalytic attack of pepsin and pancreatin on the centers of storage proteins during digestion.	Skalickova et al. [30]
Protein digestibility slightly decreased. In the Zamnè legume, the digestible protein content retracted from 76.9% (only cooked seeds) to 74.0% in fresh tempeh (48 h) and 73.8% in matured tempeh (120 h).	The decrease in digestibility and hydrolysis extent in longer fungal fermentations is attributed to the likely bioconversion of plant substrate proteins and amino acids into new fungal proteins (biomass), which have lower cellular digestibility for humans.	Drabo et al. [2]
In vitro protein digestibility significantly increased in all tested grains: in soybean, it rose from 85.84% to 95.15%; in lentil, from 78.63% to 85.02%; and in chickpea, from 81.09% to 92.63%.	The fungus *P. ostreatus* secretes proteolytic enzymes and tannases that hydrolyze proteins into smaller peptides and free amino acids. Furthermore, the intense degradation and reduction in antinutrients (tannins and phytates) limit protein cross-linking, leaving them exposed and susceptible to digestive enzyme attack.	Noori et al. [9]
The bioprocess increased the hydrolyzed protein fraction. The total extent of proteolysis after the intestinal digestion phase reached 57% in fermented lentil flour, compared to only 40% in the unfermented control flour.	Proteins undergo intense pre-hydrolysis during the fermentation process driven by the lytic mechanisms of the fungus itself. Complementarily, fermentation reduces antinutritional factors that originally exert an inhibitory role on digestive proteases.	Asensio-Grau et al. [53]
The digestibility of the cereal bars declined. The soluble protein portion in the digesta fell from 39.4 g/100 g of protein (bar with only macerated faba bean) to 34.7 g/100 g of protein (bar containing faba bean fermented with *P. ostreatus*).	The combination of fermentation with thermal treatments alters protein secondary structures (with an increase in the formation of A1 and A2 protein aggregates that hinder enzymatic access). Concurrently, browning and the Maillard reaction form melanoidins, compounds that reduce the activity of host proteolytic enzymes, such as trypsin.	Khvostenko et al. [56]
The in vitro protein digestibility (IVPD) index grew linearly with natural fermentation time. In raw white lima bean, it was 50.33%, rising to 53.17% (24 h), 55.07% (48 h), 57.84% (72 h), and reaching 58.50% after 96 h.	The fermenting microbiota performs proteolysis releasing previously retained proteins, inducing structural modifications that make them highly vulnerable to stomach enzyme action. This pathway occurs in synergistic association with the global reduction in antinutritional factors in the matrix.	Ojo et al. [38]
The protein digestibility of red kidney bean rose from 29.04% (raw matrix) and 62.05% (after cooking/pre-fermentation) to a mark of 80.97% after lactic fermentation.	The improvement in digestibility is driven by the action of microbial enzymes produced by lactic acid bacteria (LAB), which are actively responsible for cleaving intact proteins into smaller and more easily digestible peptides by the gastrointestinal tract.	Gumienna et al. [58]
The in vitro protein digestibility (IVPD) of the unfermented mixed cereal and legume beverage was 43.6%. After fermentation by lactic bacteria consortia, IVPD significantly increased, reaching levels that ranged between 48.1% and 70.1% (depending on the starter combination).	The increase in digestibility results from the strong proteolytic activity of the lactic consortia combined with the drastic reduction in antinutritional factors in the matrix. Bacterial metabolism also acts on specific phenolic compounds that originally create complexes and hinder access for digestive enzymes.	Viretto et al. [1]
The IVPD of black chickpea rose from 80% (raw dough) to 85% (spontaneous fermentation) and reached 91% after fermentation with *L. plantarum*. When applied in pasta dough, the formulation with the fermented ingredient reached 73% IVPD (against 45% for pure wheat dough).	The increment is promoted by intense proteolytic activity conducted by lactic acid bacteria coupled with the reduction in antinutritional factors (such as tannins and trypsin inhibitors), which eliminates blockages and exposes the protein structure to human digestive enzymes.	De Pasquale et al. [41]
Fermentation increased digestibility (IVPD) in all six tested legumes. Red lentil went from 72% (raw) to 83% (fermented raw) and 90% (fermented gelatinized). Chickpea rose from 80% to 91% to 94% range post-fermentation.	A combined and synergistic effect occurs between endogenous seed proteases (activated by moisture/heat) and LAB microbial peptidases, which induce intense pre-hydrolysis. In parallel, antinutritional factors that inhibit stomach proteases undergo severe degradation.	De Pasquale et al. [42]
The digestibility (IVPD) of yellow pea rose from 84.0% (raw matrix) to 87.8% after simple fermentation, reaching its maximum efficiency of 89.2% when subjected to the combined process of germination, fermentation, and pasteurization.	Bioprocess alters and weakens the grain’s storage protein structure. Massive hydrolysis of thermolabile and thermostable antinutritional compounds (such as trypsin inhibitors and tannins) also occurs, unlocking human protease attack on protein substrates.	Ma et al. [43]
Although it does not express a classic IVPD percentage, peptidomic profiling of the gastrointestinal digesta attested that tempeh produces significantly shorter protein fragments (predominance of peptides with 8 to 11 amino acids) and a lower rate of resistant domains compared to raw soybean (which generated long peptides > 13 AAs).	Fermentation via *Rhizopus oligosporus* results in global and extensive hydrolysis of soy proteins and eradicates endogenous seed protein inhibitors. This pre-digestive modification breaks blocking regions and makes peptide chains hyper-accessible to digestive juices and epithelial peptidases.	Wu et al. [23]

**Table 5:** Effect of fermentation on resistant starch, glycemic index, and proposed mechanisms of action.

Does Fermentation Increase Resistant Starch Content?	Proposed Mechanism for the Alteration of Resistant Starch	Effect of Fermentation on the Glycemic Index	Proposed Mechanism for the Alteration of the Glycemic Index	Reference
Yes. Resistant starch (RS) increased from 42.19 to 45.00 g/100 g in cowpea; from 27.51 to 56.73 g/100 g in sorghum; and from 42.38 to 46.28 g/100 g in sweet potato.	Released metabolites (such as lactic acid) cause esterification reactions in starch chains, providing steric hindrance and hindering access by digestive enzymes.	Reduction in the rapid digestion peak. The rapidly digestible starch (RDS) fraction decreased from 21.15 to 19.94 g/100 g in cowpea, and from 20.36 to 18.07 g/100 g in sorghum.	The synthesis of organic acids lowers the pH and promotes a strong inhibitory impact on amylolysis. Propionic acid also contributes by delaying gastric emptying.	Kewuyemi et al. [26]
Yes. In lentil flour subjected to bioprocessing with *Pleurotus ostreatus*, resistant starch went from 66.5% to 76.2%.	The fungus preferentially consumes the simplest carbohydrates in the matrix as a carbon source, resulting in the concentration of the resistant fraction and the modification/retrogradation of starch.	Reduction in in vitro digestion. The maximum carbohydrate hydrolysis index at 120 min decreased from 34% (control) to 24% (fermented).	The higher proportion of resistant starch imposes a slowdown in matrix degradation, avoiding abrupt glucose secretions and decreasing the glycemic load.	Asensio-Grau et al. [53]
Yes. Fermentation significantly increased the resistant starch fraction, reaching 15.0% in the bioprocessed multi-grain sample.	Does not explore the molecular physicochemical mechanism linking the change to the global bioconversion of the grain matrix (rice, legumes, and soybean) by the probiotic yeast.	Increase in rapid digestibility. RDS content increased from 60.8 to 83.9% whereas content of SDS reduced from 28.5% to 3.5%.	The yeast hydrolyzes complex carbohydrates and weakens the cell wall microstructure, making underlying carbohydrates hyper-available to digestive juices.	Banik et al. [7]
No. Resistant starch fell continuously in faba bean: from 6.12% (raw) to 4.10% (24 h), 4.69% (48 h), 5.68% (72 h), and 5.92% (96 h).	The spontaneous fermentation microbiota utilizes oligosaccharides and destroys cell wall fibers, unprotecting and disintegrating the native starch granular barrier.	Increase in the Glycemic Index (GI). The GI increased from 57.11 (raw control) to 57.18 (24 h), 62.38 (48 h), 62.67 (72 h), and 62.82 (96 h)	The profound degradation of cellular matrix fibers fully exposes the starch, accelerating the enzymatic attack and glucose absorption.	Ojo et al. [38]
Stable/Variable. In dehulled faba bean breads, RS ranged from 1.45% (raw) to 1.26% (fermented). In protein-rich faba bean, it ranged from 0.93% to 0.95%.	Resistant starch levels are primarily associated with thermogelatinization and retrogradation occurring during oven baking.	Matrix dichotomy. The Hydrolysis Index (HI) rose from 99.9% to 119.0% in dehulled faba bean, but reduced from 133.4% to 114.9% in protein-rich faba base flour.	In protein-rich matrices (gluten and faba bean), fermentation induces strong protein aggregation that restricts water availability. This prevents complete starch gelatinization in the oven, blocking attack by hydrolases.	Hoehnel et al. [25]
Yes. Resistant starch increased from 0.7 g/100 g (control) to 0.8–0.9 g/100 g in matrices fermented by lactic consortia.	The increase stems from structural physicochemical modifications that restrict enzymatic access due to the acidity of lactic processing.	Sharp drop (low GI). The HI fell from 52.0% to 24.9%. The Predictive Glycemic Index (pGI) reduced from 68.3 (high) to 53.4 (low GI).	Lactic acid bacteria release organic acids and fibers (exopolysaccharides), structuring an environment that vigorously stunts the catalytic rates of starch enzymatic digestion.	Viretto et al. [1]
Yes. Fermented black chickpea flour dough increased resistant starch retention: 1.75% (raw) to 2.81% (starter fermentation).	Bioprocess-based dynamics cause steric reorganization in flours, retrograding structures that physically prevent carbohydrases from accessing the granule center.	Progressive reduction. The pGI was reduced from 79.8 (wheat) and 78.1 (raw chickpea) to 72.6 in the final fermented matrix. The HI fell from 75% to 60%.	The structural blockade allied with the acidic environment generated by *Lactiplantibacillus plantarum* hinders fluid-cellular transport of starches and affects amylase affinity.	De Pasquale et al. [41]
Yes. Drastic elevation occurred. Black bean flour rose from 9.13% to 15.92% (when gelatinization and fermentation were combined). Red lentil flour rose from 2.22% to 4.12%.	Lactic metabolism in conjunction with heat (gelatinization) forms a retrograded resistant crystalline shield that chemically restructures the starch polymer.	Systemic drop. The HI fell in all tested flours: in black bean it fell from 68% (raw) to 62% (fermented) and 58% (gelatinized/fermented).	The high concentration of microbial organic acids slows gastric emptying and directly inhibits the catalysis of human host α-amylase enzymes.	De Pasquale et al. [42]
No (Significant reduction). In yellow pea, resistant starch suffered a massive drop from 340 g/kg (native seed) to 201 g/kg (after fermentation) and 118 g/kg (after germination, fermentation, and pasteurization).	Lactic strains preferentially consume raw starch fractions as an energy substrate, depleting the granule reserve along with cell wall lysis.	Increased glycemic response (Digestible Starch). RDS rose from 88 g/kg to 179 g/kg (fermented) and up to 258 g/kg (germinated + fermented + pasteurized).	The bioprocess combined with thermal degradation disintegrates crystallinity granules, softening the fibers. The starch becomes 100% exposed and susceptible to rapid conversion into free glucose.	Ma et al. [43]
Yes. Jack bean experienced a slight increase from 5.7% (cooked) to 6.1% after fungal tempeh fermentation.	The *Rhizopus* fungus devours the simpler (digestible) portion for its proliferation, concentrating by density the total volume of residual refractory starch in the sample.	Digestible starch reduction. The non-resistant (digestible) starch fraction dropped significantly from 41.3% to 29.6%.	The consumption of readily accessible starch-derived carbon sources by the fungus during fermentation results in the final release of less glucose in vitro in the lumen of the gastrointestinal model.	Purwandari et al. [60]

**Table 6:** Impact of different fermentation strategies on the bioaccessibility and bioavailability of minerals in pulse matrices, as evaluated by in vitro digestion models, and their underlying biophysicochemical mechanisms.

Effect of Fermentation on Mineral Bioaccessibility and/or Bioavailability in in Vitro Digestion Models	Proposed Mechanisms Involved in the Alteration of Mineral Bioaccessibility	Reference
Traditional homemade fermentation significantly decreased (26% to 47%) the total selenium (Se) content and substantially reduced gastric bioaccessible fractions in in vitro models of cereal/legume batter mixtures.	The decrease results from chemical leaching into the pre-fermentative steeping water and the short duration of the process (12 h) adopted at the domestic level, which induces the metabolic fixation of insoluble bonds that resist the action of simulated stomach digestive juices.	Khanam et al. [37]
Fermentation increased the in vitro bioaccessibility of chromium (Cr) by 9% to 18%, but significantly reduced the digestive release rates for the microminerals copper (Cu) and manganese (Mn) in raw fermented batters.	The optimization for Cr stems from partial hydrolysis mediated by the microflora; however, for Cu and Mn, the short natural fermentation time (12–14 h) prevented exogenous phytases or tannases from having sufficient time to fully cleave and digest the chelating barriers originating from the bean hull.	Kumari et al. [34]
The fermentation process with *Lactobacillus plantarum* did not promote improvements or showed inhibitory effects on the in vitro gastric bioaccessibility of Zn^2+^ and Fe^3+^ in protein concentrates.	The bacterial strain failed to excrete sufficient phytase to cleave the high levels of insoluble protein-phytate-mineral complexes of the legumes formed at acidic pH and in the cellular lumen, requiring isolated purified phytases to achieve effective atomic release.	Zhang et al. [27]
Fermentative bioprocessing significantly increased the concentration and in vitro gastric bioaccessibility of iron (Fe), manganese (Mn), magnesium (Mg), and zinc (Zn) available in the fluids after digestive simulation.	The production of metabolites, especially organic acids (e.g., lactic acid) and enzymes by lactic acid bacteria, disrupts the protective polysaccharide matrix of the grain, and acts by promoting chemical and ionic reduction in minerals such as Fe into more soluble formats for epithelial absorption.	Skalickova et al. [30]
Solid-state fermentation (tempeh) demonstrated a significant increase (173%) in dialyzability and simulated bioaccessibility of zinc (Zn) in the raw grain tested in the in vitro digestive tract.	The intense proliferation of *Rhizopus oryzae* hyphae secretes an extracellular enzymatic load (especially phytases and cellulases) that degrades the insoluble fiber complexes and actively breaks down phytic acid chains, unlocking the metal ions.	Drabo et al. [2]
Fungal conversion selectively favored the bioaccessible release of magnesium (Mg) reaching 24%, retained high levels (79%) of iron (Fe) bioaccessibility, but regressed the final absorptive rates for calcium (Ca) in vitro.	Specific metabolic modifications guided by *Pleurotus ostreatus* hyphae induce different catabolic routes, in which certain specific metals end up re-complexing, masked within the insoluble non-digestible residual structures of the medium generated during cellular fermentation.	Khvostenko et al. [56]
Microbial fermentation significantly elevated the in vitro gastrointestinal tract bioaccessibility of Fe, Mn, Cu, and Zn from 10.1% to 18.9%, in addition to actively increasing the release of selenomethionine (SeMet) forms.	The inoculated microorganism (*Bacillus subtilis* natto) possesses proteases that degrade 50 to 60% of the insoluble plant protein barrier into soluble nitrogenous compounds, converting minerals and selenoproteins formerly inactive in the matrix into active and soluble atomic forms in gastric fluids.	Zhao et al. [49]
In vitro digestion integrated with the co-cultured human cell model (Caco-2/HT29-MTX) demonstrated an expressive increase in cellular biological iron uptake (via ferritin quantification) in all evaluated tempehs compared to the temperature/coagulation treatment (tofu).	Fermentation virtually completely depletes inositol hexaphosphate (chelating phytate) due to the exogenous work of phytases activated in the metabolic cycle of *Rhizopus* fungi. The drastic elimination of this antinutrient allows the transport and absorption of soluble iron and zinc through the gastric epithelial barrier.	Auer et al. [61]
The technological integration combining ultrasound and warm fermentation (36 °C) substantially maximized (almost twofold increases) the rates in absorptive bioaccessibility and simulated in vitro release of cellular calcium and iron.	The cavitation driven by the physical ultrasound treatment causes structural disruption and strongly exposes intracellular and extracellular mineral binding sites. Such injuries actively synergize in fungus-based enzymatic fluids operated at high temperatures, hydrolyzing and releasing calcic and ferric clusters into the intestinal epithelial aqueous phases.	Harahap et al. [3]

**Table 7:** Impact of fermentation on the content and in vitro bioaccessibility/bioavailability of bioactive compounds in pulse and grain matrices.

Effect on Bioactive Compound Content in Grains	Proposed Mechanism for the Alteration of Pre-Digestion Content	Effect on Bioaccessibility/Bioavailability During in Vitro Digestion	Proposed Mechanism for the Alteration of Bioaccessibility	Reference
Total Phenolic Content (TPC) increased: in faba bean, TPC rose from 2.40 mg GAE/g (flour) to 4.05 mg GAE/g (post-fermentation). In yellow pea, pure compounds such as protocatechuic acid jumped from 31.69 μg/g (flour) to 93.38 μg/g.	Solid-state bioprocessing physically disrupts the grain, while microbial hydrolytic activities break down cell walls, releasing phytochemicals and phenolics that were previously insoluble (covalently bound to the plant matrix).	**Drastic increase.** After in vitro digestion (SGID), the bioaccessible fraction of fermented faba bean jumped to 19.14 mg GAE/g (compared to 11.07 mg GAE/g of the digested raw flour). Protocatechuic acid from pea reached massive peaks of bioaccessible release at 426.12 μg/g.	The prior structural loosening by fermentation, combined with microbial enzymatic degradation (pre-digestion), leaves phenolic compounds vulnerable. This enables human gastric proteolytic enzymes and pH fluctuations to massively extract phytochemicals into soluble fluids.	Di Stefano et al. [50]
The use of probiotic inocula in flours strongly increased the seed’s defenses. Fermented cowpea reached the highest ranges of polyphenols (TPC between 0.83 and 1.91 mg GAE/g), also leveraging the direct indices of the flavonoid taxifolin and acids (gallic, p-coumaric).	Endogenous microbial and processing-based enzymatic activities actively increase titratable acidity (TTA) and local organic acids (such as lactic and oxalic acid), which dissolves microstructures and unlocks isolated phenolic phytochemicals.	Not quantitatively evaluated.	Not applicable.	Kewuyemi et al. [26]
Biological fermentation with *Rhizopus oligosporus* produced significant strict increases (*p* < 0.05) in Total Flavonoid Content (TFC) and Total Phenolic Content (TPC). These rises accompanied a robust increase in oxidative blockage in markers such as DPPH, ABTS, and FRAP in the evaluated legumes.	The fungal matrix acts through exogenous hydrolytic enzymes and polyphenol oxidase; these degradations break down complex meshes like tannins and release smaller aromatic compounds, bioconverting phenols into their detectable free form in the flour chemistry.	Not quantitatively evaluated.	Not applicable.	Toor et al. [48]
There was an increase in Total Phenolic Content (TPC) from 2.09 to 3.2 mg Gallic Acid Equivalents (GAE)/g dry matter (DM). FRAP antioxidant capacity rose from 1.53 to 1.82 mg Trolox/g DM. DPPH and ABTS capacities remained stable.	The secretion of enzymes such as laccases (phenol-oxidases) by the fungus *Pleurotus ostreatus* degrades cell wall lignin. Cellular hydrolysis breaks down the grain matrix and promotes the release of conjugated/bound phenolic compounds.	**TPC increased, but antioxidant capacity reduced.** After digestion, the fermented flour presented a higher free TPC fraction (7.73 mg GAE/g DM) compared to the control (7.11 mg GAE/g DM). However, the antioxidant capacity of the bioaccessible fraction reduced in DPPH (6.0 to 4.7 mg Trolox/g) and FRAP (3.63 to 2.35 mg Trolox/g).	The parallel hydrolysis of proteins and starch, combined with gastric acidity, unlocks and massively solubilizes polyphenols. However, *de novo* phenolic compounds generated by fermentation present greater instability and thermolability during digestion, resulting in lower antioxidant activity.	Asensio-Grau et al. [53]
The use of lactic acid bacteria increased the TPC of chickpea flour by ~2.7 times (from 91.2 to 248.6 µg/g DM) and the total antioxidant capacity (TAC) by ~11 times. There was a marked production of free pyrogallol (123.5 µg/g), which was absent in the control.	Enzymes expressed by lactic acid bacteria (esterases, glucosidases, phenolic acid decarboxylases) promote the rupture of plant cell walls, bioconverting and releasing phenolics. Pyrogallol originates from the microbial decarboxylation of gallic acid following tannin hydrolysis.	**Strongly increased TPC.** Digestion induced a 1.3 to 4.1-fold increase in bioaccessible TPC compared to raw products. The TPC of the fermented flour’s soluble fraction reached 480.0 µg/g (versus 137.2 µg/g in the control). There was high cellular bioavailability (Caco-2 model) of pyrogallol (65%) in the basolateral fractions.	The acidic environment originating from lactic acidification previously destabilizes complex matrix conformations, facilitating subsequent phenolic solubilization. Transit through digestive fluids and enzymes releases polyphenols that had been trapped by matrix breakdown during cooking.	Chiacchio et al. [32]
In the germinated brown rice and chickpea beverage, fermentation reached peaks at 36 h. TPC rose from 123.42 to 164.37 mg GAE/100 mL and flavonoids (TFC) from 283.75 to 613.75 mg RE/100 mL. There was a jump in DPPH activity (40.98% to 81.16%), ABTS (up to 76.37%), and FRAP.	Microbial hydrolytic enzymes (such as cellulase, esterase, and β-glucosidase) actively decompose complex macromolecular compounds and cleave cell walls, converting insoluble matrices into bioavailable free polyphenolic monomers and flavonoids in the solution.	**Attenuation of antioxidant capacity.** After digestion of the 36 h fermented sample, DPPH capacity reduced from 81.16% to 58.95%, ABTS fell from 76.37% to 63.25%, and FRAP reduced from 3.44 to 0.84 µmol/L (although parameters remained above the unfermented control).	The fractional loss of antioxidant potential stems from the strong binding of soluble polyphenols to pepsin (via Van der Waals forces and hydrogen bonds) in the gastric phase, coupled with destabilization induced by the pH increase and catalysis by medium enzymes during intestinal simulation.	Wu et al. [23]
The inclusion of fermented faba bean in the bars resulted in lower TPC (2.4 mg GAE/g DM) compared to the purely macerated control (3.71 mg GAE/g DM). DPPH capacity fell from 5.7 to 4.0 mg TE/g DM, while in the ABTS assay it rose from 1.0 to 1.14 mg TE/g DM.	Fluctuations occur because, in complex baked matrices (bars), free phenolics undergo non-linear interactions (such as the formation of melanoidins under heat). Lower phenolic content relates to variation in prior metabolic tyrosine deaminations by the fungal hyphae in the matrix.	**Marked increase in TPC, but a drop in antioxidant activity.** In the fermented bar digesta, TPC bioaccessibility jumped to 11.9 mg GAE/g DM (compared to 2.4 in the raw bar). Conversely, soluble antioxidant capacities in the digesta (DPPH and FRAP) dropped abruptly.	The major leap in total phenolic bioaccessibility is due to reactions induced by the high activity of hydrolytic enzymes previously secreted during fermentation, which aid cellular dismantling. Free antioxidant activity losses arise from oxidations, antagonistic reactions, or chelation by other macromolecules generated in the intestinal lumen.	Khvostenko et al. [56]
Although total phenolics were reduced, specific compounds increased. *p*-Hydroxybenzoic acid rose from 7.71 to 48.40 µg/g and vanillic acid from 2.66 to 16.17 µg/g. Antioxidant capacity (ORAC) significantly increased from 302.69 to 508.78 mM TE/g after co-fermentation.	Microbial enzymes (such as β-glucosidases and esterases) and proteases hydrolyze cross-links in the plant cell wall matrix, releasing bound phenolic compounds and fragmenting proteins into antioxidant peptides.	**Drastic increase.** After simulated gastrointestinal digestion, the total bioaccessible phenolic content in the fermented sample grew 5-fold, from 65.91 mg GAE/g to 321.41 mg GAE/g. The inhibition of reactive oxygen species (cellular ROS) increased from 40.13% to 66.58%.	Passage through gastric and intestinal fluids promotes additional chemical modifications in phenolics and proteins. Digestive enzymes cleave larger peptides, releasing free amino acids and additional phenolic compounds with superior antioxidant activity compared to their precursors.	Bautista-Expósito et al. [36]
Lactic acid fermentation of the red kidney bean base increased polyphenols by 13% (from 3.33 to 3.76 mg GAE/g DM) and antioxidant activity by 72% (from 7.03 to 12.11 mg TE/g DM). Snacks with fermented marjoram addition (RBM) reached peaks of 3.66 mg GAE/g DM and 10.63 mg TE/g DM.	Enzymes such as proteases and α-amylases produced by lactic acid bacteria aid in dismantling the legume cellular framework, synthesizing or releasing free phenolic compounds, which potentiates the final product’s antioxidant reactivity.	**Continuous increase.** Polyphenols and antioxidant activity reached maximum values during the large intestine stage. In the snack with marjoram (RBM), phenolics jumped from 2.85 (pre-digestion) to 5.61 mg GAE/g of digesta (at 18 h), and antioxidant activity rose from 5.92 to 28.82 mg TE/g.	The symbiotic intestinal microbiota drives strong biotransformation of polyphenols. Intestinal bacteria produce enzymes like β-glucosidase, which intensively metabolizes remaining phenolic compounds, converting them into secondary metabolites with high free antioxidant capacity.	Gumienna et al. [58]
Soy kefir exhibited a reduction in glycosides and a significant increase in free bioactive forms (aglycones). Daidzein concentration rose from 0.12 mg/g to 0.74 mg/g and genistein increased from 0.05 mg/g to 1.14 mg/g.	The component microorganisms of kefir grains produce and secrete the enzyme β-glucosidase during fermentation, responsible for hydrolyzing glycoside conjugates and bioconverting them into corresponding active aglycone forms.	**Maintenance of high bioavailability.** In the digested fraction, free aglycones remained at significantly higher levels in the digested kefir (daidzein: 0.77 mg/g; genistein: 0.59 mg/g) compared to the digested unfermented soy solution (0.09 and 0.15 mg/g, respectively).	Unlike isoflavones in their glycosidic form (which do not cross the membrane via passive diffusion), aglycones are absorbed more easily and rapidly by the epithelium. The prior accumulation of these aglycones via fermentation preserves high accessibility in digestive juices and ensures greater cellular bioavailability.	Luo et al. [40]
Prior to digestion, tempeh fermentation at 36 °C significantly elevated Total Phenolic Content (TPC) from ~12 (raw soy) to ~35 mmol GAE/g DM and Total Flavonoid Content (TFC) from ~5 to ~38 mmol QE/g DM. The combination of dual ultrasound and fermentation at 36 °C (USC-36) maximized the values, reaching a TPC of ~51 mmol GAE/g DM and a TFC of ~41 mmol QE/g DM.	The increase in fermentation temperature intensifies microbial metabolism and the expression of hydrolytic enzymes (such as β-glucosidase), which cleave glycosides releasing aglycones. Ultrasound pretreatment causes cavitation and mechanical microjets that disrupt cell walls and protein-polysaccharide matrices, exposing previously insoluble phytochemicals.	**Significant increase in bioaccessible flavonoids.** After in vitro digestion, TFC bioaccessibility in the USC-36 group reached a maximum of ~7%, representing an almost twofold increase compared to other treatments (which obtained~4%). Bioaccessible TPC remained in the range of ~30 mmol GAE/g DM in USC-36.	Ultrasound cavitation treatment combined with fermentation acidification and hydrolysis causes the loosening of the macromolecular matrix and cell wall rupture. This weakens the original plant binding forces, facilitating structural solubilization and the transport of flavonoids to the aqueous phase during enzymatic cleavage in the digestive tract.	Harahap et al. [3]

**Table 8:** Effect of microbial fermentation on the degradation of antinutritional factors (phytates and tannins) in pulses, and their respective proposed biological mechanisms.

Does Fermentation Alter Phytate Content?	Proposed Mechanism for Phytate Alteration	Does Fermentation Alter Tannin Content?	Proposed Mechanism for Tannin Alteration	Reference
Yes. A progressive decrease in phytic acid occurred, reaching a minimum residual level of 1.05 mg/100 g at the end of bioprocessing.	Cellular hydrolysis actively promoted by microbial metabolism throughout the fermentation and steeping time breaks the resistance and bonds of the phytic acid molecule.	**Yes.** There was a drastic reduction. In unfermented flour (bran), the content fell from 19.64 mg/100 g to 1.38 mg/100 g in 24 h. In the fermented whole grain, the level reduced to 2.03 mg/100 g in 72 h.	Acidic conditions and microbiological action during the fermentation period promote the degradation and lysis of compounds, inactivating the ability of tannins to complex proteins.	Sobowale et al. [52]
Yes. The process eliminated up to 91.3% of phytic acid in wheat flour, with suppression rates exceeding 88% in barley and mung bean matrices.	*Lactobacillus* spp. bacterial strains actively excrete the phytase enzyme into the medium, which acts catalytically by cleaving and removing phosphate groups from phytic acid.	Not evaluated.	Not applicable.	Rajani et al. [28]
Yes. Fungal biotransformation significantly reduced phytate networks, reaching reductions of up to 55% in the case of the soybean matrix.	The inoculated fungus (*Rhizopus oligosporus*) synthesizes and excretes high concentrations of the endogenous phytase enzyme, specialized in hydrolyzing phytic acid in the solid state.	**Yes.** Levels were significantly altered, with reductions for most matrices and fluctuations (increases) reported for chickpea and pigeon pea (exact numerical values not textually reported).	The reduction stems from the degradation of complexes by polyphenol oxidase and/or tannase enzymes produced by the fungus. Punctual increases may derive from the hydrolysis of larger bonds that expose new free soluble tannins.	Toor et al. [48]
Yes. There was a significant drop and elimination of 64.50% of total phytate content, decaying from a baseline of 20.17 mg/100 g to only 7.16 mg/100 g in the final matrix.	The probiotic yeast (*Saccharomyces cerevisiae*) responds to nutrient restrictions by releasing potent volumes of the hydrolase enzyme (extracellular phytase) into the broth, which cleaves the phytate complex.	Not evaluated.	Not applicable.	Banik et al. [7]
Not evaluated.	Not applicable.	**Yes.** Fermentation reported significant reductions in insoluble tannin content, dropping to 6.02 mg TAE/g in cowpea flour and to 5.84 mg TAE/g in sorghum.	Tannin suppression is based on microbial hydrolysis executed by tannin acyl hydrolase enzymes, which are widely secreted by lactic acid bacteria.	Kewuyemi et al. [26]
Yes. A constant reduction occurred. In raw whole bean it was 174.37 mg/100 g, falling to 83.65 mg/100 g at 48 h and reaching 47.16 mg/100 g at the end of 96 h of bioprocessing.	The decrease is a direct consequence of leaching into the process water, coupled with intense biological and enzymatic activities activated by the endogenous microflora during the natural fermentation time.	**Yes.** Tannins fell from 125.16 mg/100 g in the raw matrix to 69.32 mg/100 g at 48 h and reached 43.26 mg/100 g after 96 h of continuous fermentation.	The reduction is based on the same principle: dilution in the aqueous matrix followed by progressive biodegradation of the toxic molecule by the hydrolytic enzymes of the local microbiota.	Adeyeye et al. [31]
Yes. The original content (0.397 g/100 g) decayed to 0.329 g/100 g using *Aspergillus oryzae* and reduced even more drastically (0.214 g/100 g) with the use of *Rhizopus oligosporus*.	The mechanism involves the abundant secretion of phytases by filamentous fungi during solid-state fermentation, enzymes that cleave phytic acid, with *R. oligosporus* being the most aggressive in degradation.	**Yes.** Condensed tannins were almost eliminated: from 1.56 mg CE/g (flour) to traces of 0.02 mg CE/g (*A. oryzae*) and complete elimination (0.00 mg CE/g) with *R. oligosporus*.	The strong hydrolysis of condensed tannin chains and insoluble protein complexes occurs due to the excretion of active tannase enzymes by the applied fungi.	Gautheron et al. [20]
Yes. Incubation generated 10% losses in soybean, 4% in red bean, and an expressive 28% decrease in bioprocessed mung bean flour.	Water-soluble compounds are lost in natural leaching, while the intense pH drop increases acidity, which promotes and activates the endogenous and microbial degrading phytases themselves inside the seeds.	**Yes.** The decrease was generalized, reporting reductions of 44% in soybean levels, 58% in red bean, and 18% in laboratory-fermented mung bean.	The microbiota acts by producing proteolytic enzymes that break the strong bonds of the tannin-protein complex. In parallel, polyphenol oxidase and tannin acyl hydrolase are activated, which biochemically destroy the tannin.	Nadhifa et al. [4]
Yes. Phytate decayed from 9.55 to 7.93 mg/g in lentils; from 6.67 to 5.60 mg/g in chickpea, and from 6.06 to 4.92 mg/g in soybean matrices.	The fungus acts by producing and inducing phytases in the moist solid matrix that dismantle the plant’s antinutritional constituents and successfully alter the original organic composition of the samples.	**Yes.** Bitterness fell from 1.76 to 1.27 mg GAE/g in lentils; from 0.74 to 0.55 mg GAE/g in chickpea, and from 0.52 to 0.37 mg GAE/g in processed soybean flour.	The attenuation is specifically attributed to the metabolism of the *Pleurotus ostreatus* mushroom, which produces reactions with tannase activity, invariably leading to the physical elimination of tannins in cellular tissues.	Noori et al. [9]
Yes. Fermentation dropped the levels even further. The residue (0.13–0.16 g/100 g) left over from cooking regressed significantly to levels between 0.06 and 0.13 g/100 g of phytate.	The strong catalytic activity stems from a direct correlation with fungal phytase production, which gains broad access to the already softened lupin substrate after pretreatment (debittering).	**Yes.** Fermentation caused a small complementary decrease. Previous values (154–204 mg/100 g) dropped to margins between 144.48 and 185.86 mg/100 g of retained tannins.	The breakdown of this protein-complexing biomolecule in the grain is directly credited to the organic microbiological secretion of the tannase enzyme by *Rhizopus oligosporus* hyphae.	Villacrés et al. [51]
Yes. Continuous progressive reduction occurred. In raw white faba bean flour, the content was 19.05 mg/100 g, decaying to 17.44 mg/100 g in 24 h and reaching a minimum mark of 13.20 mg/100 g after 96 h of natural fermentation.	The reduction is attributed to leaching into the steeping water combined with enzymatic degradation catalyzed by phytase enzymes secreted by the local fermenting microorganisms of the seed flora.	**Yes.** Tannins fell significantly from 35.29 mg/100 g in the unfermented matrix to limits of 19.05 mg/100 g in the processed flour after the 96 h fermentation cycle.	Tannin suppression is based on leaching into the soaking water and cellular hydrolysis operated by degrading enzymes synthesized by the microbiota during fermentation progression.	Ojo et al. [38]
Yes. A drastic and significant reduction in the antinutrient occurred. Phytic acid in raw jack bean was 325.47 mg/100 g and abruptly reduced to 102.23 mg/100 g in fermented seeds.	The authors associate the expressive nullification of toxic levels actively with the intrinsic degradation operated by the biological metabolic process during solid-state fermentation.	**Yes.** There was a minimal, non-statistically significant reduction. Levels in the raw matrix of 0.35 g/100 g slightly altered to 0.34 g/100 g after biological fermentation.	The timid nullification towards fermentation occurs due to the complexity of extraction in the tissue, with biotransformation being less effective in breaking tannin structures in this legume compared to combined thermomechanical treatments (autoclaving).	Arise et al. [57]
Yes. Phytic acid was reduced by about 40%, falling from 69.2 mg/100 mL in the unfermented matrix to 35.5 mg/100 mL in the fermented snack.	Lactic acid bacteria (LAB) provide exogenous microbial phytases that actively degrade and dephosphorylate the phytic acid molecule.	**Yes.** Condensed tannin concentration decreased by approximately 90%, going from 1.61 mg/100 mL to 0.21 mg/100 mL.	Lactic acid bacteria strains directly hydrolyze tannin-protein complexes through their expressive tannase enzyme activity.	Pontonio et al. [21]
Yes. Phytate concentration fell from 350.11 mg/100 g (cooked grain) to 143.98 mg/100 g after 120 h in a salt-sugar solution, and to 109.07 mg/100 g in a salt-only solution.	The reduction is attributed to the activity of the phytase enzyme from the grains themselves and from fermenting microorganisms (LAB), as well as the passive diffusion of water-soluble phytates.	**Yes.** Tannins decayed from 274.77 mg/100 g to 97.07 mg/100 g in the salt-sugar solution and to 73.67 mg/100 g in the salt-exclusive solution (reductions of 64.7% and 73.19%).	The tannase enzyme hydrolyzes the polyphenolic compounds of tannin complexes. The process was more effective in the salt solution due to the higher pH, which favors optimal tannase activity.	Kitum et al. [44]
Yes. After fermentation with *Lactobacillus casei*, phytate levels in the red kidney bean (RKB) beverage fell 71% (to 7.23 mg/L). In the green mung bean (GMB) beverage, the reduction was 49% (to 11.23 mg/L).	Degradation occurs due to the utilization and metabolization of these compounds by probiotic bacteria during beverage fermentation.	**Yes.** Tannins in the red kidney bean (RKB) beverage were reduced by 42% (reaching 0.28 mg/L) after probiotic fermentation.	The decrease results from microbial catabolism, where the probiotic culture utilizes polyphenolic fractions, reducing the antinutritional load.	Chaturvedi et al. [45]
Yes. Fungal bioprocessing raised the phytate content from 1.50% in the raw seed to 4.06% at 24 h, 3.50% at 48 h, and 3.09% at 72 h of fermentation.	The variations are attributed to the biochemical reactions of the fungus *Rhizopus oligosporus*, which can induce the synthesis or release of these components from the seed matrix.	**Yes.** Tannins increased from 0.38% (raw seed) to 0.50% (24 h), 0.49% (48 h), and 0.41% after 72 h of fermentation.	Enzymatic fermentation with *Rhizopus oligosporus* alters the organic composition, leading to biosynthesis and increased availability of phenolic compounds and tannins in the matrix.	Ezegbe et al. [59]
Yes. The initial content of 1.6 g/100 g fell to 1.3 g/100 g under fermentation with *Pleurotus eryngii* and achieved the greatest reduction (0.6 g/100 g) with *Trametes versicolor* after 5 days (63% loss).	The fungal mycelium produces phytases that hydrolyze phytic acid into bioavailable forms of phosphorus (orthophosphates and myo-inositol) to support cell growth.	**Yes.** The content of 5.8 mg CE/g in raw beans fell to the range of 1.1–2.0 mg CE/g with *P. eryngii* and 0.8–1.2 mg CE/g using *T. versicolor*.	Fungi excrete the tannase enzyme which destroys the tannin molecule, although enzymes such as cellulase can release polyphenols locked in the plant cell wall.	Li et al. [5]

**Table 9:** Systematic matrix of core influencing factors driving experimental inconsistencies and knowledge gaps in fermented legumes.

Core Influencing Factor	Mechanism of Action	Observed Experimental Inconsistency/Contradiction	Target Knowledge Gap
Substrate Properties & Matrix Recalcitrance	Varied protein-dense vs. starch-dense ratios; presence of thick, fibrous seed coats.	Lactic fermentation reduces the hydrolysis index in high-protein fava bean matrices but increases it in low-protein, dehulled counterparts [25]. Fungal strains may paradoxically elevate quantified phytates/tannins in recalcitrant seeds due to partial cleavage of complex polymers [48,59].	Lack of transcriptomic profiling to match specific microbial strains to individual botanical varieties.
Initial Moisture & Physical State	Submerged Fermentation (SmF) drives high fluid mobility; Solid-State Fermentation (SSF) creates heterogeneous porous beds.	Fungal SSF effectively reduces starch hydrolysis in high-moisture gels but exerts no significant hypoglycemic effects when applied to dry-baked matrices [6].	Missing predictive stability modeling regarding multi-strain consortia dynamics under fluctuating scale-up conditions.
pH Adjustment & Organic Acid Accumulation	Dropping medium pH activates native seed hydrolases but can cause structural denaturation of microbially secreted enzymes.	Rapid acidification in salt-sugar environments drops pH below 4.0, denaturing fungal tannases and limiting tannin degradation compared to buffered, salt-only solutions [44].	Scarcity of high-resolution chromatographic profiling (LC-MS/MS) to track exact degraded intermediates (IP_4_, IP_3_).
Thermal & Mechanical Pretreatments	Fine milling breaks cell wall barriers; intensive autoclaving/cooking triggers starch gelatinization.	Pairing fermentation with autoclaving or severe pasteurization collapses starch crystallinity, causing digestible starch availability to surge and overriding prior biochemical modulation [43].	Failure of static in vitro models (e.g., INFOGEST) [2,30,49,53] to replicate complex in vivo endocrine, hormonal, and brush-border transport dynamics.

## Data Availability

No new data were created or analyzed in this study. Data sharing is not applicable to this article.

## Supplementary Materials

The following supporting information can be downloaded at: https://www.mdpi.com/article/10.3390/foods15142483/s1, Supplementary Material S1: Reproducible R workflow for automated screening and textual data preparation. References [62,63,64] are cited in Supplementary Materials.
