# Supplementary material for: Biotechnological Modulation of Legumes via Fermentation: Impacts on Nutrient Bioaccessibility, Glycemic Index, and Antinutrients—A Scoping Review
Source: Foods. 2026 Jul 13;15(14):2483. doi: 10.3390/foods15142483 (PMC13409413; doi:10.3390/foods15142483)
Supplement: Supplementary file 1 [file foods-15-02483-s001.zip › foods-4375680-supplementary.pdf]

Supplementary Data 1

Supplementary Material S1. Reproducible R Workflow for Automated Screening and Textual Data Preparation

Description

This supplementary material provides the R workflow used to support the reproducible screening and refinement of the bibliographic database included in the scoping review. The script was applied after duplicate removal in EndNote 20.0.1 and before manual title and abstract screening by the independent reviewers.

The automated procedures included:

- exclusion of review articles;
- exclusion of books and book chapters;
- exclusion of conference abstracts and proceedings;
- exclusion of studies related to silage;
- exclusion of studies related to farm animal feeding and animal nutrition.

The workflow was implemented in R version 4.4.1 (R Foundation for Statistical Computing, Vienna, Austria) using the RStudio integrated development environment.

R Script Used for Automated Filtering

```
# ============================================================
# SCOPING REVIEW – AUTOMATED FILTERING WORKFLOW
# ============================================================
# R version: 4.4.1
# Software: RStudio
# Purpose:
# Automated refinement of bibliographic records prior to
# manual screening.
# ============================================================


# ------------------------------------------------------------
# Load packages
# ------------------------------------------------------------
```

```r
library(tidyverse)

library(dplyr)

library(stringr)

library(readr)


# ------------------------------------------------------------

# Import database

# ------------------------------------------------------------

# Replace with the exported file from EndNote or database


references <- read_csv("references.csv")


# ------------------------------------------------------------

# Standardize text fields

# ------------------------------------------------------------


references <- references %>%

  mutate(

    title = str_to_lower(title),

    abstract = str_to_lower(abstract),

    keywords = str_to_lower(keywords),

    document_type = str_to_lower(document_type)

  )


# ------------------------------------------------------------

# Remove review papers

# ------------------------------------------------------------
```

```
review_terms <- c(
  "review",
  "systematic review",
  "meta-analysis",
  "scoping review",
  "literature review"
)

references_no_review <- references %>%
  filter(
    !str_detect(title, str_c(review_terms, collapse = "|")),
    !str_detect(document_type, str_c(review_terms, collapse = "|"))
  )

# ----------------------------------------------------------
# Remove books and book chapters
# ----------------------------------------------------------

book_terms <- c(
  "book",
  "book chapter",
  "encyclopedia"
)

references_no_books <- references_no_review %>%
  filter(
    !str_detect(document_type, str_c(book_terms, collapse = "|"))
```

```
  )

# ---------------------------------------------------------
# Remove conference abstracts/proceedings
# ---------------------------------------------------------

abstract_terms <- c(
  "conference abstract",
  "meeting abstract",
  "proceeding",
  "conference paper"
)

references_no_abstracts <- references_no_books %>%
  filter(
    !str_detect(document_type, str_c(abstract_terms, collapse = "|"))
  )

# ---------------------------------------------------------
# Remove studies related to silage
# ---------------------------------------------------------

silage_terms <- c(
  "silage",
  "ensiling",
  "forage"
)
```

```
references_no_silage <- references_no_abstracts %>%
  filter(
    !str_detect(title, str_c(silage_terms, collapse = "|")),
    !str_detect(abstract, str_c(silage_terms, collapse = "|")),
    !str_detect(keywords, str_c(silage_terms, collapse = "|"))
  )

# ----------------------------------------------------------
# Remove farm animal feeding studies
# ----------------------------------------------------------

animal_feed_terms <- c(
  "animal feed",
  "feedlot",
  "ruminant",
  "broiler",
  "swine",
  "pig",
  "cattle",
  "cow",
  "goat",
  "sheep",
  "poultry",
  "livestock",
  "animal nutrition",
  "feed supplementation"
)
```

```
references_final <- references_no_silage %>%
  filter(
    !str_detect(title, str_c(animal_feed_terms, collapse = "|")),
    !str_detect(abstract, str_c(animal_feed_terms, collapse = "|")),
    !str_detect(keywords, str_c(animal_feed_terms, collapse = "|"))
  )

# ---------------------------------------------------------

# Export final screened database

# ---------------------------------------------------------

write_csv(references_final, "references_filtered.csv")

# ---------------------------------------------------------

# Summary of filtering steps

# ---------------------------------------------------------

cat("Initial records:", nrow(references), "\n")
cat("After removing reviews:", nrow(references_no_review), "\n")
cat("After removing books:", nrow(references_no_books), "\n")
cat("After removing abstracts/proceedings:", nrow(references_no_abstracts), "\n")
cat("After removing silage studies:", nrow(references_no_silage), "\n")
cat("Final records included:", nrow(references_final), "\n")
```

Notes on Reproducibility

The filtering workflow was designed to increase reproducibility and transparency during the study selection stage of the scoping review. Automated exclusion procedures were applied conservatively and subsequently verified during manual screening performed independently by two reviewers.

The keyword-based filtering strategy was used only as a support tool for database refinement and did not replace manual eligibility assessment.

Software and Packages

- R version 4.4.1 (R Foundation for Statistical Computing, Vienna, Austria)
- RStudio (Posit Software, Boston, MA, USA)
- tidyverse
- dplyr
- stringr
- readr

Suggested Citation

R Core Team. R: A language and environment for statistical computing. Vienna: R Foundation for Statistical Computing; 2024.

Wickham H, Averick M, Bryan J, et al. Welcome to the tidyverse. Journal of Open Source Software. 2019;4(43):1686.

Wickham H, François R, Henry L, Müller K, Vaughan D. dplyr: A Grammar of Data Manipulation. R package version 1.1.4; 2023.
